# CSPT-SleepNet: An Automatic Sleep Staging Framework Integrating Center-Driven Sequence Supervision and Multimodal Pyramid Token Encoding

**DOI:** 10.3390/brainsci16101108

**Published:** 2026-10-14

**Authors:** Xiaoqin Lian, Peiyi Wang, Chao Gao, Yelan Wu

**Affiliations:** 1 School of Computer and Artificial Intelligence, Beijing Technology and Business University, Beijing 102488, China; lianxq@th.btbu.edu.cn (X.L.); 2431061049@st.btbu.edu.cn (P.W.); wuyel@th.btbu.edu.cn (Y.W.); 2 Key Laboratory of Industrial Internet and Big Data, China National Light Industry, Beijing Technology and Business University, Beijing 102488, China

**Keywords:** sleep staging, polysomnography, center-driven sequence supervision, multimodal feature fusion, hierarchical multi-scale representation, inter-epoch modeling

## Abstract

**Highlights:**

**What are the main findings?**

- Center-driven sequence supervision improved center-epoch classification and produced more balanced transition behavior.
- The Multimodal Pyramid Token Encoder improved sleep stage classification by hierarchically learning and integrating multi-scale temporal representations.

**What are the implications of the main findings?**

- Weak supervision using labels from neighboring epochs can offer stage-informed guidance for contextual learning while preserving the center epoch as the primary target.
- Hierarchical multi-scale representation learning provides a structured way to integrate temporal information within each modality before cross-modal interaction.

**Abstract:**

**Background:** Automatic sleep staging is fundamental to sleep architecture analysis and the diagnosis of sleep disorders. Sleep staging requires jointly modeling complementary information across modalities, multi-scale physiological features, and temporal relationships across consecutive epochs. In target epoch prediction from consecutive epochs, non-target epochs primarily provide contextual information, but their ground-truth labels remain underused as auxiliary supervision. **Methods:** We propose CSPT-SleepNet, an automatic sleep staging framework integrating center-driven sequence supervision with multimodal pyramid token encoding. A Multimodal Pyramid Token Encoder extracts hierarchical multi-scale features from electroencephalogram, electrooculogram, and electromyogram signals, followed by intra-modal, cross-modal, and inter-epoch modeling for center-epoch classification. During training, the center epoch receives primary classification supervision, while non-center epochs receive weaker auxiliary supervision based on their ground-truth labels, providing stage-informed contextual support for the prediction target. **Results:** In subject-independent cross-validation on Sleep-EDF-20, Sleep-EDF-78, and ISRUC-S3, CSPT-SleepNet achieved accuracy (ACC)/macro F1 (MF1) scores of 87.93%/83.53%, 85.81%/80.87%, and 84.06%/82.86%, respectively. In a separate controlled comparison on Sleep-EDF-20, center-driven sequence supervision improved ACC and MF1 by 0.60 and 0.91 percentage points, respectively, over equal-weight sequence supervision. The full model has 3.418 million trainable parameters and requires 397.10 million floating-point operations for an input sequence of nine 30-s epochs. **Conclusions:** CSPT-SleepNet provides an effective framework for center-epoch sleep staging from consecutive multimodal inputs. Nevertheless, N1 recognition remains challenging, with F1 scores ranging from 55.3% to 62.7% across the three datasets.

## 1. Introduction

Sleep is a fundamental physiological process that sustains cognitive function and physiological homeostasis. Insufficient sleep can impair core executive functions, including working memory, inhibitory control, and cognitive flexibility [1]. By characterizing the composition and temporal progression of sleep stages, sleep staging provides an important basis for analyzing sleep architecture, assessing sleep quality, and supporting the diagnosis of sleep disorders [2,3]. In clinical practice, sleep specialists score polysomnography (PSG) recordings according to the Rechtschaffen and Kales (R&K) rules [4] or the guidelines of the American Academy of Sleep Medicine (AASM) [5]. The recordings are divided into consecutive 30s epochs, and sleep specialists assign a sleep stage label to each epoch. In the commonly used five-stage setting, sleep is classified into Wake (W), three non-rapid eye movement (NREM) stages (N1, N2, and N3), and rapid eye movement (REM) sleep [5]. However, manual epoch-by-epoch scoring requires substantial time and specialized expertise, and is subject to inter-rater variability, with particularly low agreement for N1 [6,7]. Therefore, accurate, efficient, and reproducible automatic sleep staging is important for large-scale sleep research and clinical sleep analysis [2,3].

**Figure 1:**
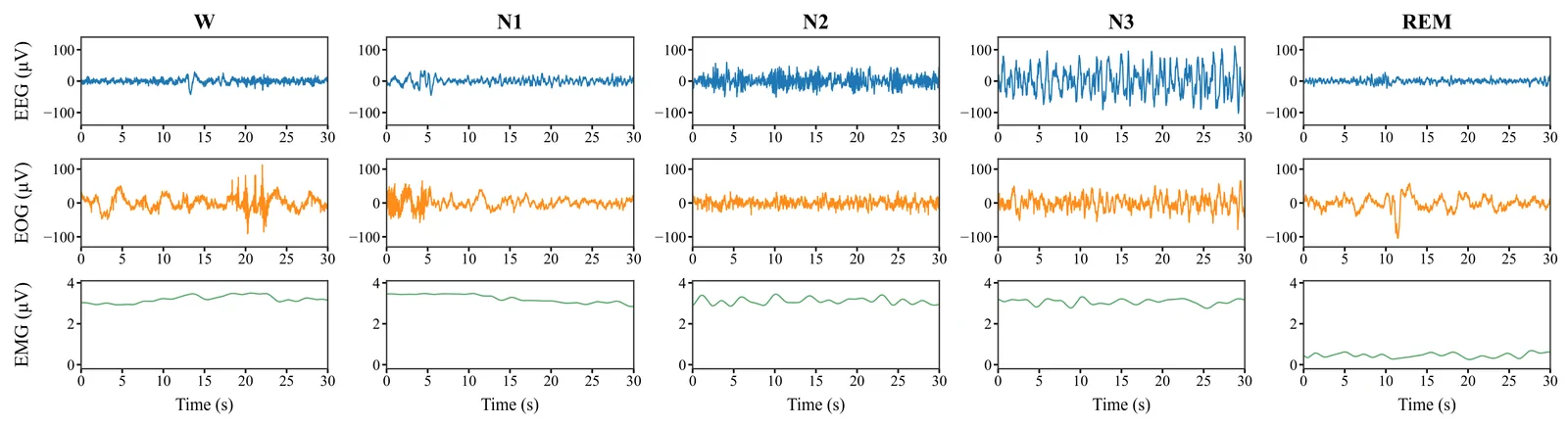
Thirty-second EEG, EOG, and EMG waveforms across the five sleep stages. The displayed epochs were randomly selected from the Sleep-EDF-20 dataset.

PSG recordings typically include synchronized electroencephalogram (EEG), electrooculogram (EOG), and electromyogram (EMG) signals. EEG provides information on brain rhythms and characteristic waveforms, EOG captures potentials related to eye movements, and EMG reflects muscle activity and tone. These signals provide complementary information for sleep stage classification [8,9]. Recent multimodal biomedical studies have also highlighted the integration of physiologically distinct signals according to task-specific objectives, combining information from different sources to support physiological state assessment and diagnostic support [10,11]. The complementary roles of different modalities in sleep staging can be illustrated using the waveform examples shown in Figure 1. N3 generally shows more prominent slow wave activity, providing a characteristic EEG cue for stage identification. N1 and REM can show similar EEG activity, with cues from eye movements and chin muscle tone providing additional information for their discrimination [5]. In addition, sleep-related rhythms and characteristic waveforms occur over different durations and frequency ranges, while consecutive epochs exhibit temporal continuity and dependencies associated with stage transitions [12,13,14]. Therefore, effective automatic sleep staging requires the joint representation of multi-scale physiological features, the integration of complementary multimodal information, and the modeling of temporal relationships across consecutive epochs.

Existing methods commonly use multi-scale architectures to capture sleep-related patterns across temporal scales. Early approaches, such as DeepSleepNet [15] and AttnSleep [16], employ parallel convolutional branches with different receptive fields to capture short-term local waveforms and longer-term rhythmic variations. In contrast, SleePyCo [12] and SleepFC [17] aggregate features across network depths into hierarchical multi-scale representations, thereby establishing information flow across scales. Recent studies have extended multi-scale modeling to multimodal PSG. MMS-SleepNet [18] and MMTSleepNet [19] extract multi-scale features from EEG, EOG, and EMG through modality-specific branches, while CAT-Sleep [20] and SleepMamba [21] further combine multi-scale representations with cross-modal interaction. However, these approaches primarily focus on extracting representations at multiple resolutions and integrating information across modalities, while explicit hierarchical relationships among different scales remain less explored. Hierarchical architectures establish cross-scale connections through progressive feature propagation, while repeated downsampling may weaken short-term local information. Therefore, multimodal PSG modeling requires multi-scale representations with hierarchical relationships and must also balance receptive field expansion with the preservation of relevant information.

After obtaining multi-scale representations for each modality, intra-modal and cross-modal relationships need to be further modeled to form an epoch-level multimodal representation that integrates physiological cues from multiple sources. Simple feature concatenation or fixed fusion strategies can combine different signals, but have limited ability to capture cross-modal dependencies. To promote multimodal feature interaction, SalientSleepNet [22] extracts salient waveforms from different modalities using separate branches and adaptively aggregates informative features through multimodal attention. Transformer approaches, such as MultiChannelSleepNet [23] and MIIT-DAL [24], model multimodal relationships by constructing joint multichannel representations or progressively integrating intra-modal and inter-modal information. More recently, state space architectures have extended this direction. SleepMamba [21] integrates multiresolution feature learning with dual-axis state-space modeling to capture dependencies within and across modalities, and employs bidirectional temporal Mamba to model long-range relationships across epochs. These recent developments demonstrate increasing attention to the joint modeling of multimodal and temporal information. Nevertheless, multi-scale feature representation, intra-modal relationship modeling, cross-modal interaction, and inter-epoch context modeling still need to be effectively coordinated to form a clearly structured information processing pipeline.

Sleep stage sequences typically exhibit continuity and regularity in stage transitions. Around stage transitions, the target epoch may contain physiological characteristics of adjacent stages, leading to greater overlap in discriminative cues across stages [13,14]. Therefore, temporal relationships between adjacent epochs should be exploited to provide cues about sleep stage evolution for target epoch classification [25]. Existing methods typically take multiple consecutive epochs as input and can be broadly categorized into target epoch prediction and joint sequence prediction according to their prediction targets. Target epoch prediction methods, such as IITNet [26], STDP-GCN [27], TBSTSleepNet [28], and SleepMFormer [29], use consecutive epochs to classify a predefined target epoch. Non-target epochs mainly provide contextual information for feature modeling, while their ground-truth labels are typically not used for direct classification supervision. By contrast, joint sequence prediction methods, including SeqSleepNet [30], XSleepNet [31], TransSleep [13], and Cross-Modal Transformer [32], simultaneously predict multiple input epochs, allowing their ground-truth labels to contribute to model optimization jointly. All epochs are treated as classification targets, typically without prioritizing a particular epoch. However, using labels from multiple epochs does not necessarily require treating them as joint classification targets. SleepRWKVNet [33] employs bidirectional interactive RWKV for efficient multimodal feature interaction and estimates Markov transition probabilities between sleep stages from subject-specific label sequences during training, and uses them to adjust the classification loss of individual epochs. The latter illustrates an alternative way in which label-derived information can participate in model optimization through explicit transition priors. Such a strategy, however, relies on statistically estimated inter-stage transition relationships. Therefore, an open question remains as to whether, within a target epoch prediction framework, non-target ground-truth labels can be used to strengthen the stage semantics of the corresponding epochs and better support target epoch classification, without treating all input epochs as equally important classification targets or introducing an explicit transition prior.

Motivated by these considerations, we propose CSPT-SleepNet. The main contributions are summarized as follows:

- We introduce a center-driven sequence supervision strategy for automatic sleep staging with consecutive epochs as input and the center epoch as the prediction target. Center-epoch classification is the primary objective, while non-center epochs receive weaker auxiliary classification supervision. This strategy preserves the dominant role of center-epoch supervision while using the ground-truth labels of non-center epochs to strengthen the contextual support for center-epoch classification.
- We design a Multimodal Pyramid Token Encoder (MPTE) for multimodal PSG. It combines hierarchical multi-scale convolution, learnable inter-level downsampling, and a fine-grained supplementary branch to construct modality-specific token representations from EEG, EOG, and EMG by integrating sleep-related features across multiple scales. These representations serve as inputs to subsequent modeling stages.
- We develop a hierarchical information processing pipeline comprising intra-modal token context modeling, cross-modal interaction, and inter-epoch context modeling. It progressively aggregates information within each modality and integrates complementary cross-modal information, while modeling temporal relationships across consecutive epochs to yield hierarchical representations for center-epoch classification.
- We conduct subject-independent cross validation on Sleep-EDF-20, Sleep-EDF-78, and ISRUC-S3. Comprehensive experiments evaluate CSPT-SleepNet from multiple perspectives and further characterize information allocation at the inter-epoch, cross-modal, and intra-modal levels.

The remainder of this paper is organized as follows. Section 2 presents the proposed CSPT-SleepNet and its training objective. Section 3 describes the experimental setup and evaluation protocol. Section 4 reports the experimental results and analyses. Section 5 provides further discussion of the results and the limitations of the study. Finally, Section 6 concludes the paper.

## 2. Proposed Method

### 2.1. Problem Formulation

For the *i*-th epoch of a whole-night PSG recording, the multimodal input is $X_{i}=\left\{x_{i}^{m}|m\in M\right\}$, with M={EEG,EOG,EMG}. For modality *m*, $x_{i}^{m}\in R^{C_{m}\times T}$ contains $C_{m}$ channels and *T* samples. Sleep stages belong to C={W,N1,N2,N3,REM}, where C=|C|=5, and the label for epoch *i* is $y_{i}\in C$. To incorporate context around the target epoch, we construct a sequence of S=2K+1 consecutive epochs centered on epoch *t*, expressed as $X_{t}=\left(X_{t-K},\ldots ,X_{t},\ldots ,X_{t+K}\right)$, with labels $Y_{t}=\left(y_{t-K},\ldots ,y_{t},\ldots ,y_{t+K}\right)$. Position *j* corresponds to epoch t−K+j−1 in the whole-night recording, where j∈{1,…,S}. The relative temporal offset of position *j* from the center epoch is denoted by $\Delta_{j}=j-c$, where $\Delta_{j}\in \left\{-K,\ldots ,0,\ldots ,K\right\}$. The center position c=K+1 corresponds to the target epoch *t*, termed the center epoch. Other epochs are non-center epochs. In this study, S=9, K=4, and c=5.

The model learns a parameterized function $f_{\theta }$ that maps the input sequence $X_{t}$ to the final logit sequence $Z_{t}^{\mathrm{final}}=f_{\theta }\left(X_{t}\right)\in R^{S\times C}$, where θ denotes the learnable parameters. The final logit at the center position receives primary classification supervision and is used to obtain the sleep stage prediction ${\hat{y}}_{t}$ for the center epoch, while the final logits at non-center positions receive weak auxiliary supervision during training.

### 2.2. Overview of the Proposed Framework

**Figure 2:**
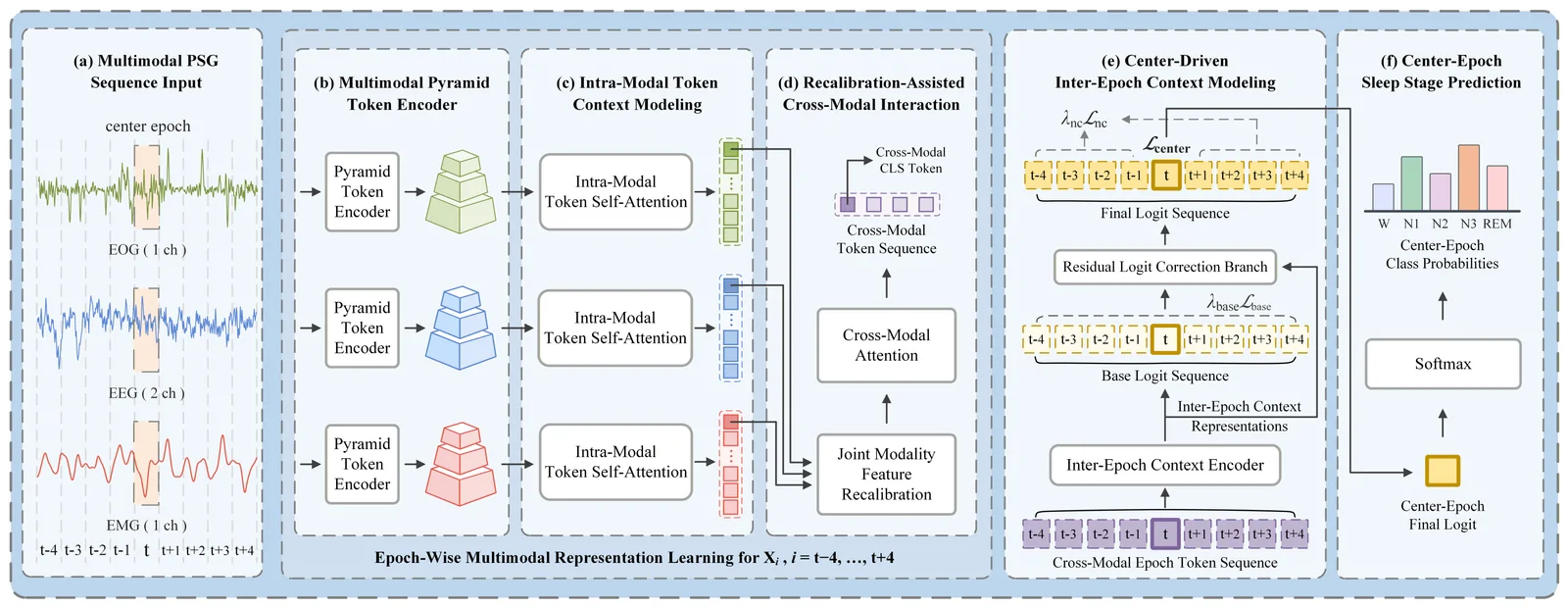
Overall architecture of the proposed CSPT-SleepNet. Highlighted tokens denote modality CLS tokens and cross-modal CLS tokens. Bold borders indicate the center position in the inter-epoch and output sequences. Solid arrows indicate the forward flow of information, whereas dashed arrows in panel (**e**) indicate the training supervision paths.

As shown in Figure 2, CSPT-SleepNet takes EEG, EOG, and EMG signals from *S* consecutive 30s epochs as input and predicts the sleep stage of the center epoch. For each epoch, the Multimodal Pyramid Token Encoder extracts multi-scale tokens for each modality, followed by intra-modal token context modeling to obtain modality-level representations. These representations undergo Joint Modality Feature Recalibration and are then passed to cross-modal attention to model cross-modal relationships and form a cross-modal epoch representation. The within-epoch encoding pipeline is applied at all relative positions in the input window using structurally identical but independently parameterized branches. Inter-epoch context modeling then captures temporal relationships across the *S* epochs and produces a sequence representation integrating multi-scale, multimodal, and inter-epoch contextual information. At each position, a classification head maps the epoch representation to base logits, which together form the base logit sequence. A lightweight residual logit correction branch predicts an additional correction that is added to the base logits to form the final logit sequence. The final logit at the center position determines the sleep stage of the center epoch.

During training, the classification loss on the final logit at the center position serves as the primary optimization objective, while non-center final logits receive weaker auxiliary supervision using the ground-truth labels of their corresponding epochs. Together, these losses form the center-driven sequence supervision. This differentiated supervision is consistent with recent sequential learning studies in which weak supervisory information supports temporal representation learning [34], while different positions are allowed to contribute unequally to the training objective [35]. In addition to the differentiated supervision applied to the final logit sequence, an auxiliary classification loss is applied to the base logit sequence to preserve the stage-discriminative capability of the uncorrected outputs and provide supervised base outputs for learning the residual logit corrections.

### 2.3. Multimodal Pyramid Token Encoder

Physiological features associated with different sleep stages vary in duration and local morphology [5]. Models therefore need to extract and organize sleep-related features at different temporal resolutions. Conventional parallel multi-scale convolutions typically process the same input through independent branches with different temporal receptive fields, but lack progressive feature propagation and integration across scales [15,16]. Inspired by the hierarchical representation of feature pyramids [12,14], we design a Multimodal Pyramid Token Encoder (MPTE) to build hierarchical multi-scale representations with progressive cross-scale propagation.

**Figure 3:**
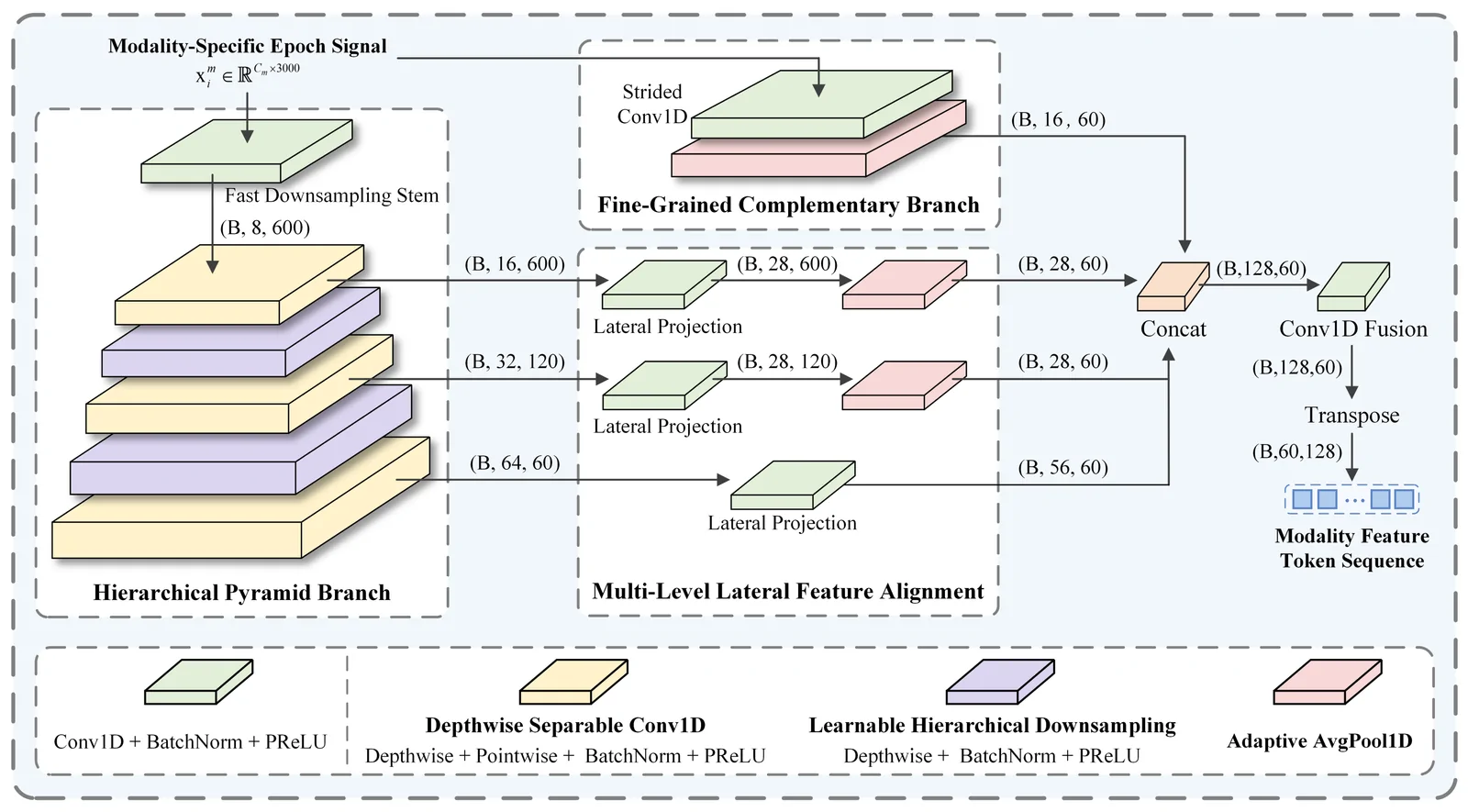
Architecture of a modality-specific encoder in the Multimodal Pyramid Token Encoder. Tensor dimensions are shown as (B,C,L), denoting batch size, channels, and temporal length, respectively. $C_{m}$ is the number of input channels for modality *m*.

MPTE consists of three structurally identical modality-specific pyramid token encoders with separate parameters, independently encoding EEG, EOG, and EMG signals. As shown in Figure 3, each encoder comprises the hierarchical pyramid branch, fine-grained supplementary branch, and multi-level lateral feature alignment module. The multi-level features from the hierarchical pyramid branch are laterally aligned, concatenated with features from the fine-grained supplementary branch, and fused into a fixed-length modality-specific feature token sequence.

For modality *m* of the *i*-th epoch in the input sequence, the signal $x_{i}^{m}$ first passes through an initial downsampling stem for feature mapping, reducing the temporal length and forming a base representation for subsequent hierarchical extraction. Three depthwise separable convolutional blocks then progressively extract hierarchical features along a hierarchical pyramid pathway, with learnable downsampling between adjacent levels. The downsampling performs local aggregation and temporal compression independently within each channel without directly mixing information across channels, while cross-channel feature mapping is performed by the pointwise convolutions in the subsequent depthwise separable convolutional blocks. This process is expressed as

(1) $$\begin{array}{c} F_{i,0}^{m}=\phi_{0}^{m}\left(x_{i}^{m}\right),\;F_{i,1}^{m}=\phi_{1}^{m}\left(F_{i,0}^{m}\right),\\ F_{i,l}^{m}=\phi_{l}^{m}\left[D_{l-1}^{m}\left(F_{i,l-1}^{m}\right)\right],\;l\in \left\{2,3\right\}. \end{array}$$

where $F_{i,l}^{m}$ is the feature map at pyramid level *l*, $\phi_{0}^{m}\left(\cdot \right)$ denotes feature mapping by the initial downsampling stem, $\phi_{l}^{m}\left(\cdot \right)$ denotes the *l*-th depthwise separable convolution block, and $D_{l-1}^{m}\left(\cdot \right)$ denotes learnable inter-level downsampling via depthwise 1D convolution.

In our implementation, each depthwise separable convolutional block consists of a depthwise 1D convolution and a pointwise 1D convolution for extracting local temporal features within each channel and mapping features across channels, respectively. The initial downsampling stem uses a 1D convolution with a kernel size and stride of 5 to produce a base representation of 8 channels and length 600. The three depthwise separable blocks have kernel sizes of 5, 5, and 3, with 16, 32, and 64 output channels, respectively. The two learnable inter-level downsampling layers use depthwise convolutions with kernel-stride pairs (5,5) and (3,2), reducing subsequent temporal lengths to 120 and 60. Each convolutional block is followed by batch normalization and PReLU activation.

The fine-grained supplementary branch extracts a shallow local representation directly from the original modality signal to complement information weakened by progressive downsampling. The computation is expressed as

(2) $$F_{i,f}^{m}={\mathrm{AAP}}_{N_{p}}\left[\phi_{f}^{m}\left(x_{i}^{m}\right)\right]\in R^{C_{f}\times N_{p}},$$

where ${\mathrm{AAP}}_{N_{p}}\left(\cdot \right)$ denotes adaptive average pooling to length $N_{p}$, and $\phi_{f}^{m}\left(\cdot \right)$ represents the fine-grained feature mapping. It consists of a 1D convolution with a kernel size and stride of 25, batch normalization, and PReLU activation, and produces $C_{f}=16$ output channels. $N_{p}$ denotes the aligned temporal length across branches and is set to $N_{p}=60$. The convolution reduces the temporal length from 3000 to 120, after which ${\mathrm{AAP}}_{N_{p}}\left(\cdot \right)$ further adjusts it to $N_{p}=60$, matching the temporal length of the deepest pyramid level.

For multi-level lateral feature alignment, each pyramid level first undergoes lateral channel projection and then matches the deepest level in temporal length, $N_{p}$. The first two levels are aligned from lengths of 600 and 120 to 60 using 1D adaptive average pooling, while the third retains its original temporal length. The aligned features are given by

(3) $$P_{i,l}^{m}=A_{l}\left[U_{l}^{m}\left(F_{i,l}^{m}\right)\right],\;l\in \left\{1,2,3\right\},$$

where $U_{l}^{m}\left(\cdot \right)$ represents the lateral channel projection at level *l*, and $A_{l}\left(\cdot \right)$ represents the corresponding temporal alignment. $A_{1}\left(\cdot \right)$ and $A_{2}\left(\cdot \right)$ use 1D adaptive average pooling to align the first two levels to $N_{p}$, while $A_{3}\left(\cdot \right)$ is an identity mapping. After lateral projection, the output channels of the three pyramid levels are allocated in a 1:1:2 ratio, giving deeper features greater channel capacity. Each projection uses a 1D convolution with a kernel size of 1 to adjust the channel number while preserving the temporal order.

After multi-level lateral feature alignment, we concatenate the features from the three pyramid levels with the fine-grained supplementary features along the channel dimension. The concatenated features are then convolutionally fused to integrate hierarchical and fine-grained information. We transpose the fused representation to obtain the modality feature token sequence $H_{i}^{m}$ for subsequent attention modeling:

(4) $$H_{i}^{m}={\left\{\psi^{m}\left[\mathrm{Concat}\left(P_{i,1}^{m},P_{i,2}^{m},P_{i,3}^{m},F_{i,f}^{m}\right)\right]\right\}}^{⊤}\in R^{N_{p}\times d},$$

where Concat(·) indicates concatenation along the channel dimension. $\psi^{m}\left(\cdot \right)$ comprises a 1D convolution with a kernel size of 1, batch normalization, and PReLU activation, and fuses the features from the pyramid levels with the fine-grained features into a *d*-dimensional embedding. The transpose rearranges the fused representation from $d\times N_{p}$ to $N_{p}\times d$, yielding $N_{p}$ tokens, each with embedding dimension *d*. We set d=128, so MPTE encodes $x_{i}^{m}$ as $H_{i}^{m}\in R^{60\times 128}$, which is then passed to intra-modal token context modeling.

### 2.4. Intra-Modal Token Context Modeling

MPTE encodes EEG, EOG, and EMG into separate modality feature token sequences that integrate hierarchical features across temporal resolutions with shallow local information. However, these tokens have not yet established global dependencies, and each sequence lacks a compact representation for that modality. Therefore, intra-modal token context modeling is applied separately for the three modality feature token sequences.

**Figure 4:**
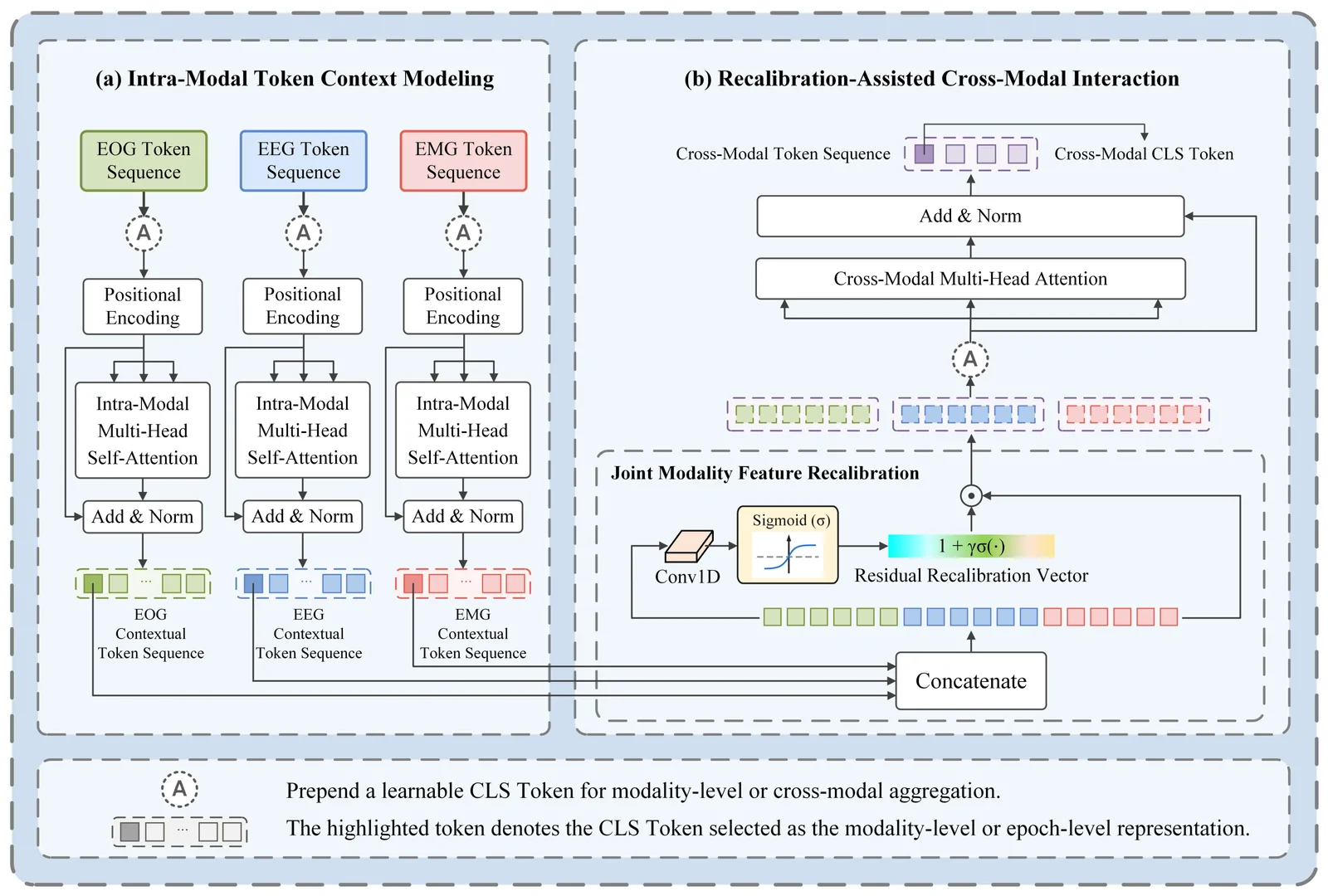
Architecture of intra-modal token context modeling and cross-modal interaction. (**a**) Intra-modal self-attention models token relationships within each modality after adding a modality CLS token and positional encoding. (**b**) The updated modality CLS tokens undergo Joint Modality Feature Recalibration followed by cross-modal attention to form a cross-modal epoch token.

Following the Cross-Modal Transformer design [32], a learnable modality CLS token is prepended to each feature token sequence and interacts with all tokens through intra-modal multi-head self-attention to aggregate modality-level contextual information. As shown in Figure 4a, EEG, EOG, and EMG use structurally identical but independently parameterized branches to model intra-modal token relationships.

For epoch *i*, modality *m* is represented by the token sequence $H_{i}^{m}\in R^{N_{p}\times d}$. A learnable modality CLS token $c^{m}\in R^{d}$ is prepended before positional encoding and dropout:

(5) $${\tilde{H}}_{i}^{m}=\mathrm{Dropout}\left(\left[c^{m}∥H_{i}^{m}\right]+E^{\mathrm{pos}}\right)\in R^{(N_{p}+1)\times d},$$

where ‖ denotes concatenation along the token dimension, and $E^{\mathrm{pos}}\in R^{(N_{p}+1)\times d}$ is the standard sinusoidal positional encoding matrix. Since self-attention does not explicitly preserve token order, sinusoidal positional encoding is added to the full sequence of the modality CLS token and feature tokens to indicate their positions.

The token sequence is then updated by intra-modal multi-head self-attention. For the *r*-th head, the input is projected into query, key, and value vectors, and scaled dot-product attention yields a contextual output. Outputs from all heads are concatenated along the feature dimension and linearly projected to obtain the intra-modal attention output [36]:

(6) $$\begin{array}{cc} Q_{i,r}^{m} & ={\tilde{H}}_{i}^{m}W_{m,r}^{Q}+b_{m,r}^{Q},\\ K_{i,r}^{m} & ={\tilde{H}}_{i}^{m}W_{m,r}^{K}+b_{m,r}^{K},\\ V_{i,r}^{m} & ={\tilde{H}}_{i}^{m}W_{m,r}^{V}+b_{m,r}^{V}. \end{array}$$

(7) $${\mathrm{head}}_{i,r}^{m}=\mathrm{Softmax}\left(\frac{Q_{i,r}^{m}{\left(K_{i,r}^{m}\right)}^{⊤}}{\sqrt{d_{h}}}\right)V_{i,r}^{m}.$$

(8) $${\mathrm{MHA}}_{m}\left({\tilde{H}}_{i}^{m}\right)=\mathrm{Concat}\left({\mathrm{head}}_{i,1}^{m},\ldots ,{\mathrm{head}}_{i,n_{h}}^{m}\right)W_{m}^{O}+b_{m}^{O}.$$

where $n_{h}$ is the number of attention heads, and $d_{h}=d/n_{h}$ is the dimension of each head. $W_{m,r}^{Q}$, $W_{m,r}^{K}$, and $W_{m,r}^{V}\in R^{d\times d_{h}}$ denote the query, key, and value projection matrices of the *r*-th head for modality *m*, with bias vectors $b_{m,r}^{Q}$, $b_{m,r}^{K}$, and $b_{m,r}^{V}\in R^{d_{h}}$. $W_{m}^{O}\in R^{d\times d}$ and $b_{m}^{O}\in R^{d}$ denote the output projection matrix and bias after head concatenation. Intra-modal multi-head self-attention assigns weights based on query-key relevance across subspaces. The corresponding values are then weighted and aggregated to obtain token representations that incorporate intra-modal contextual information.

After the multi-head self-attention output is obtained, dropout, a residual connection, and layer normalization are applied. The CLS token at the beginning of the sequence is then extracted as the modality-level representation:

(9) $$\begin{array}{c} {\bar{H}}_{i}^{m}=\mathrm{LN}\left[{\tilde{H}}_{i}^{m}+\mathrm{Dropout}\left({\mathrm{MHA}}_{m}\left({\tilde{H}}_{i}^{m}\right)\right)\right]\in R^{(N_{p}+1)\times d},\\ h_{i,\mathrm{cls}}^{m}={\bar{H}}_{i}^{m}\left[1,:\right]\in R^{d}. \end{array}$$

where LN(·) denotes layer normalization. After the residual update, each feature token preserves its own representation while incorporating context from other tokens in the same modality. The leading token $h_{i,\mathrm{cls}}^{m}$ summarizes the modality-level context and serves as the global representation of the token sequence. The three modality CLS tokens are then used for cross-modal interaction.

### 2.5. Cross-Modal Interaction Assisted by Joint Modality Feature Recalibration

After intra-modal token context modeling, EEG, EOG, and EMG are represented by modality CLS tokens that aggregate the contextual information of each modality. We apply cross-modal attention to capture their interactions and introduce Joint Modality Feature Recalibration (JMR) before cross-modal interaction to moderately adjust the feature-wise responses of the modality CLS tokens. As shown in Figure 4b, the three modality CLS tokens are first concatenated along the feature dimension. A lightweight 1D convolution followed by a sigmoid generates feature-wise recalibration weights, which are applied to the joint representation through residual scaling [37]. This process is formulated as:

(10) $$\begin{array}{c} g_{i}=\mathrm{Concat}\left(h_{i,\mathrm{cls}}^{\mathrm{EOG}},h_{i,\mathrm{cls}}^{\mathrm{EEG}},h_{i,\mathrm{cls}}^{\mathrm{EMG}}\right)\in R^{M\times d},\\ {\hat{g}}_{i}=g_{i}⊙\left\{1+\gamma \sigma \left[\mathrm{Conv1D}\left(g_{i}\right)\right]\right\}. \end{array}$$

where M=3 denotes the number of input modalities, σ(·) is the sigmoid activation function, ⊙ denotes element-wise multiplication, and γ controls the recalibration strength and is set to 0.3. Since the sigmoid output is bounded between 0 and 1, this setting constrains the residual scaling factor 1+γσ(·) to the range from 1 to 1.3, allowing moderate feature recalibration while retaining the original joint representation as the main component. The recalibrated joint representation ${\hat{g}}_{i}$ is then split into three *d*-dimensional modality representations, ${\hat{h}}_{i,\mathrm{cls}}^{\mathrm{EOG}}$, ${\hat{h}}_{i,\mathrm{cls}}^{\mathrm{EEG}}$, and ${\hat{h}}_{i,\mathrm{cls}}^{\mathrm{EMG}}$, where ${\hat{h}}_{i,\mathrm{cls}}^{m}\in R^{d}$.

After Joint Modality Feature Recalibration, the three recalibrated modality CLS tokens and a learnable cross-modal CLS token form the modality-level token sequence $U_{i}^{\mathrm{cm}}$. Multi-head self-attention then performs cross-modal interaction [36]:

(11) $$\begin{array}{c} U_{i}^{\mathrm{cm}}=\left[c^{\mathrm{cm}}∥{\hat{h}}_{i,\mathrm{cls}}^{\mathrm{EOG}}∥{\hat{h}}_{i,\mathrm{cls}}^{\mathrm{EEG}}∥{\hat{h}}_{i,\mathrm{cls}}^{\mathrm{EMG}}\right]\in R^{(M+1)\times d},\\ {\bar{U}}_{i}^{\mathrm{cm}}=\mathrm{LN}\left[U_{i}^{\mathrm{cm}}+\mathrm{Dropout}\left({\mathrm{MHA}}_{\mathrm{cm}}\left(U_{i}^{\mathrm{cm}}\right)\right)\right]\in R^{(M+1)\times d},\\ h_{i}^{\mathrm{cm}}={\bar{U}}_{i}^{\mathrm{cm}}\left[1,:\right]\in R^{d}. \end{array}$$

where $c^{\mathrm{cm}}\in R^{d}$ is a learnable cross-modal CLS token and ${\mathrm{MHA}}_{\mathrm{cm}}\left(\cdot \right)$ denotes cross-modal multi-head self-attention. Cross-modal attention enables input-dependent interaction between the cross-modal CLS token and the three modality CLS tokens. The updated cross-modal CLS token $h_{i}^{\mathrm{cm}}$ aggregates multimodal information from the current epoch and serves as the cross-modal epoch token.

The procedure yields one cross-modal epoch token for each epoch in the input window. These tokens are arranged chronologically into a cross-modal epoch token sequence, whose inter-epoch contextual relationships are modeled in the following section.

### 2.6. Center-Driven Inter-Epoch Context Modeling and Center-Epoch Prediction

At this point, each epoch in the input window is represented by a cross-modal token. The tokens from all positions are ordered chronologically and passed to the inter-epoch context module to model sleep stage continuity and transitions between adjacent epochs. This process yields base and final logit sequences for the entire input window. The final logit vector at the center position is used to predict the sleep stage of the center epoch.

**Figure 5:**
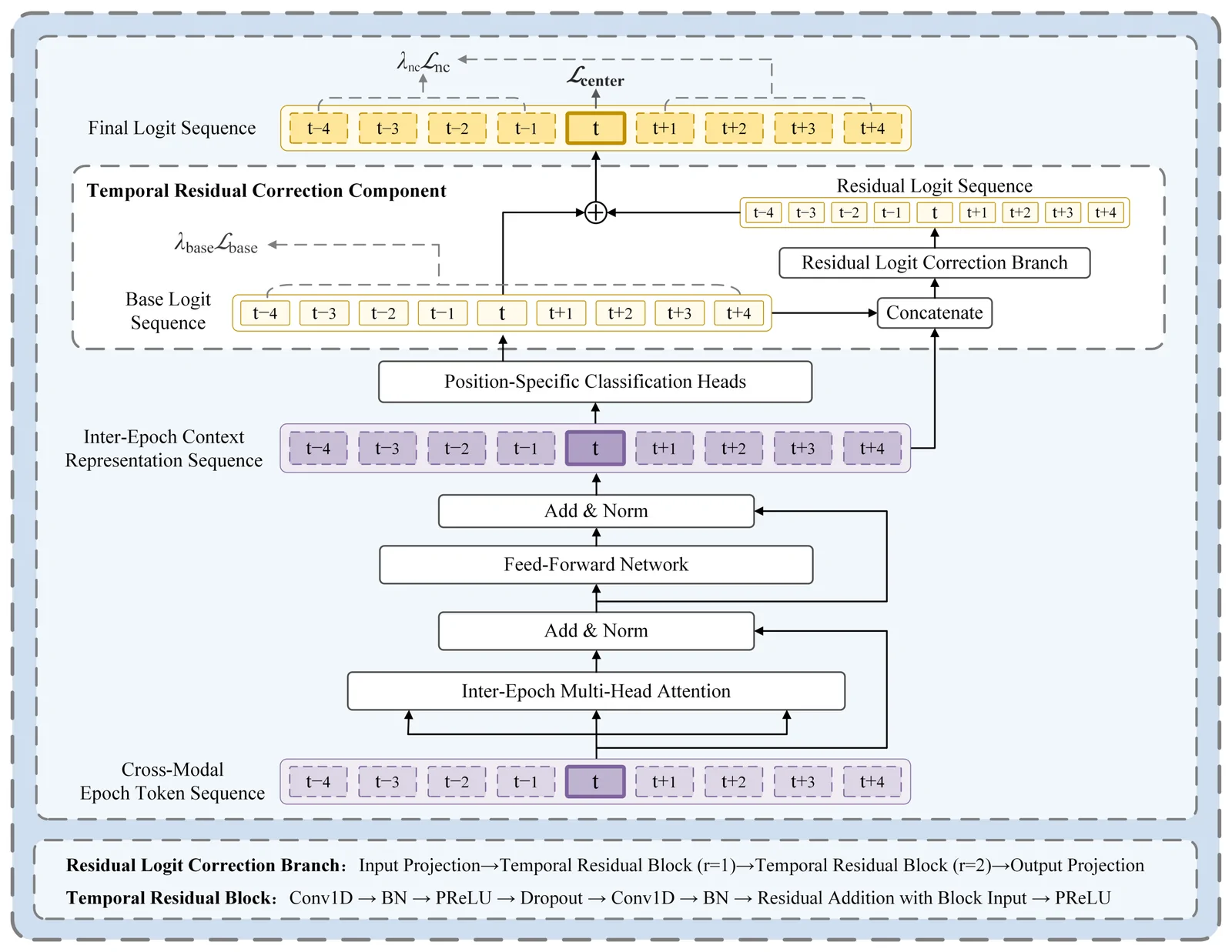
Architecture of inter-epoch context modeling and center-driven sequence supervision. Bold borders indicate the center position. Solid arrows indicate forward information flow. Dashed arrows denote the training loss terms $L_{\mathrm{center}}$, $\lambda_{\mathrm{nc}}L_{\mathrm{nc}}$, and $\lambda_{\mathrm{base}}L_{\mathrm{base}}$ applied to the center final logit, non-center final logits, and base logit sequence, respectively. The dashed box denotes the TRC component, whose residual logit correction branch and temporal residual blocks are expanded below.

As shown in Figure 5, the cross-modal epoch token sequence is processed by the inter-epoch context encoder to incorporate information from neighboring epochs, after which the classification head is applied at each position to generate the base logit sequence. The residual logit correction branch combines the resulting sequence representation with the base logits to supplement the base predictions and obtain the final logit sequence for all positions. This branch, together with auxiliary supervision on the base logit sequence, forms the Temporal Residual Correction (TRC) component.

The input window is centered on epoch *t*, with length S=2K+1 and center index c=K+1. After the cross-modal interaction described in Section 2.5, the epoch at each position *j* is represented by a cross-modal epoch token. These tokens are stacked chronologically to form the input sequence for inter-epoch context modeling:

(12) $$E_{t}^{\mathrm{cm}}=\mathrm{Stack}\left(h_{t,1}^{\mathrm{cm}},\ldots ,h_{t,S}^{\mathrm{cm}}\right)\in R^{S\times d}.$$

where $E_{t}^{\mathrm{cm}}$ is the resulting cross-modal epoch token sequence, and Stack(·) denotes stacking along the sequence dimension. For position *j*, $h_{t,j}^{\mathrm{cm}}\in R^{d}$ is generated by the corresponding within-epoch encoding branch. The *S* branches have the same architecture but independent parameters, and each branch is associated with a fixed relative offset $\Delta_{j}=j-c$ from the center epoch. This position-specific parameterization allows different relative positions to learn their own feature transformations while providing implicit relative-position information. Accordingly, no additional explicit positional encoding is used at the inter-epoch level.

The inter-epoch context encoder jointly models the cross-modal epoch tokens at all window positions using multi-head self-attention and a feed-forward network:

(13) $$H_{t}^{\mathrm{ie}}=F_{\mathrm{ie}}\left(E_{t}^{\mathrm{cm}}\right)={\left[h_{t,1}^{\mathrm{ie}},\ldots ,h_{t,S}^{\mathrm{ie}}\right]}^{⊤}\in R^{S\times d},$$

where $F_{\mathrm{ie}}\left(\cdot \right)$ is the inter-epoch context encoder, consisting of multi-head self-attention, residual connections, layer normalization, and a feed-forward network. $h_{t,j}^{\mathrm{ie}}$ is the epoch representation at position *j*. Self-attention exchanges information across positions in the window, so each representation retains information from its own epoch while incorporating information related to sleep stages from neighboring epochs. The resulting sequence reflects sleep stage continuity and transition dependencies within the window.

Each sequence position has a distinct temporal role within the centered context window according to its fixed relative offset from the center epoch and its corresponding position-specific encoding branch. Structurally identical but independently parameterized classification heads are used for position-specific decoding of these contextualized representations. The resulting base logit sequence is

(14) $$Z_{t}^{\mathrm{base}}=\mathrm{Stack}\left(f_{\mathrm{cls}}^{\left(1\right)}\left(h_{t,1}^{\mathrm{ie}}\right),\ldots ,f_{\mathrm{cls}}^{\left(S\right)}\left(h_{t,S}^{\mathrm{ie}}\right)\right)\in R^{S\times C},$$

where $f_{\mathrm{cls}}^{\left(j\right)}\left(\cdot \right)$ is the classification head for position *j*. The base logit sequence is obtained directly from the epoch representations, used as input to the residual logit correction branch, and receives auxiliary supervision during training.

The residual logit correction branch in TRC further refines the outputs after inter-epoch context modeling. It concatenates the inter-epoch context representation sequence and the base logit sequence along the feature dimension, generates a residual logit sequence, and adds it element-wise to the base logit sequence:

(15) $$\begin{array}{c} \Delta Z_{t}=f_{\mathrm{corr}}\left(\mathrm{Concat}\left(H_{t}^{\mathrm{ie}},Z_{t}^{\mathrm{base}}\right)\right),\\ Z_{t}^{\mathrm{final}}=Z_{t}^{\mathrm{base}}+\Delta Z_{t}. \end{array}$$

where $f_{\mathrm{corr}}\left(\cdot \right)$ denotes the residual logit correction mapping, $\Delta Z_{t}\in R^{S\times C}$ is the residual logit sequence, and $Z_{t}^{\mathrm{final}}\in R^{S\times C}$ is the final logit sequence.

As shown in Figure 5, the residual logit correction branch supplements the base classification results using local relationships among neighboring epochs. Two temporal residual blocks with dilation rates of 1 and 2 progressively expand the receptive field along the epoch sequence, providing multi-range local context for correction. Residual connections within the blocks preserve the input features and stabilize information propagation.

During inference, only the center-epoch final logit vector is taken from $Z_{t}^{\mathrm{final}}$ and converted by Softmax into class probabilities to obtain the prediction for the current window.

### 2.7. Center-Driven Training Objective

To align the training objective with the center-epoch prediction task, we apply differentiated supervision to classification outputs at different input positions. The final logit of the center epoch receives primary classification supervision, whereas those of non-center epochs receive auxiliary classification supervision with a lower weight, together constituting the center-driven sequence supervision. The non-center epochs are directly constrained by their own ground-truth stage labels, which serve as auxiliary supervision during training, and remain secondary in optimization. They provide stage-informed contextual support for center-epoch prediction while preserving the primary role of center-epoch classification. In addition, the complete base logit sequence receives auxiliary supervision to constrain the classification output before temporal residual correction.

Given the final logit sequence $Z_{t}^{\mathrm{final}}$ and the base logit sequence $Z_{t}^{\mathrm{base}}$ obtained in Section 2.6, the final and base logits at position *j* are denoted by $z_{t,j}^{\mathrm{final}}=Z_{t}^{\mathrm{final}}\left[j,:\right]$ and $z_{t,j}^{\mathrm{base}}=Z_{t}^{\mathrm{base}}\left[j,:\right]$. For the input window centered on epoch *t*, the ground-truth sleep stage label at position *j* is $y_{t,j}=y_{t-K+j-1}$, with $y_{t,c}=y_{t}$ for the center position c=K+1. Let $l_{\mathrm{CE}}\left(z,y\right)$ denote the cross-entropy loss between logit z and ground-truth label *y*. To account for the uneven distribution of sleep stages, we use its class-weighted form during training to strengthen the supervision of less represented stages.

Center-epoch classification is the primary optimization objective for the current window. Its classification loss on the final logit is defined as

(16) $$L_{\mathrm{center}}=l_{\mathrm{CE}}\left(z_{t,c}^{\mathrm{final}},y_{t}\right).$$

For non-center epochs, the final logit at each position is constrained by the ground-truth label of the corresponding epoch. The non-center auxiliary loss is defined as

(17) $$L_{\mathrm{nc}}=\frac{1}{S-1}\sum_{\begin{array}{c} j=1\\ j\neq c \end{array}}^{S}l_{\mathrm{CE}}\left(z_{t,j}^{\mathrm{final}},y_{t,j}\right).$$

The loss is averaged over all S−1 non-center positions and enters the total objective with a lower weight, so center-epoch classification remains the primary optimization target. Each non-center output is supervised by the ground-truth stage label of its corresponding epoch, helping the associated representation retain stage-discriminative information and provide stage-informed support for center-epoch classification.

To constrain the classification outputs before temporal residual correction, auxiliary classification supervision is applied to the complete base logit sequence:

(18) $$L_{\mathrm{base}}=\frac{1}{S}\sum_{j=1}^{S}l_{\mathrm{CE}}\left(z_{t,j}^{\mathrm{base}},y_{t,j}\right).$$

This loss acts directly on the complete base logit sequence to preserve the capacity of the uncorrected outputs to discriminate sleep stages. As an accompanying training constraint for TRC, it allows the residual logit correction branch to learn supplementary corrections from supervised base outputs.

Combining the three losses above, the total training objective is defined as

(19) $$L=L_{\mathrm{center}}+\lambda_{\mathrm{nc}}L_{\mathrm{nc}}+\lambda_{\mathrm{base}}L_{\mathrm{base}},$$

where $\lambda_{\mathrm{nc}}$ and $\lambda_{\mathrm{base}}$ control the contributions of non-center auxiliary supervision and auxiliary supervision on the base logits, respectively. In our implementation, both coefficients are set to 0.3. The value of $\lambda_{\mathrm{nc}}$ keeps the supervision on neighboring epochs weaker than the center-epoch objective, so that these epochs provide explicit stage guidance without shifting the main optimization focus away from center-epoch classification. The value of $\lambda_{\mathrm{base}}$ provides a mild constraint on the predictions before residual correction, helping the base outputs retain stage-discriminative information while allowing the correction branch to focus on refinement.

## 3. Experimental Setup

### 3.1. Datasets and Preprocessing

We evaluate the proposed model on three public sleep staging datasets, Sleep-EDF-20, Sleep-EDF-78, and ISRUC-S3, to assess its classification performance across different data settings [38,39,40].

**Sleep-EDF-20 and Sleep-EDF-78:** Both datasets were derived from the Sleep Cassette (SC) subset of the public PhysioNet Sleep-EDF Expanded database [38,39]. Sleep-EDF-78 contains 153 whole-night recordings from 78 healthy subjects (37 males and 41 females) aged 25–101 years, whereas Sleep-EDF-20 corresponds to a commonly used 20-subject subset of the larger Sleep-EDF-78 cohort, comprising 39 whole-night PSG recordings from 20 healthy subjects (10 males and 10 females) aged 25–34 years. Each PSG recording includes Fpz-Cz EEG, Pz-Oz EEG, horizontal EOG, submental EMG, and other physiological signals. All recordings were annotated epoch by epoch by sleep experts according to the Rechtschaffen and Kales (R&K) scoring standard, with each 30 s epoch labeled as stage 0, stage 1, stage 2, stage 3, stage 4, REM, MOVEMENT, or UNKNOWN [4].

**ISRUC-S3:** This dataset, released by Khalighi et al., contains 10 whole-night PSG recordings from 10 healthy subjects (9 males and 1 female) aged 30–58 years, with one recording per subject [40]. Each recording includes 6 EEG channels (F3-A2, C3-A2, O1-A2, F4-A1, C4-A1, and O2-A1), 2 EOG channels (LOC-A2 and ROC-A1), 3 EMG channels (Chin EMG, left leg movements, and right leg movements), 1 ECG channel, and respiratory and blood oxygen signals. Two sleep specialists independently annotated each recording epoch by epoch according to the AASM criteria [5], labeling each 30 s epoch as W, N1, N2, N3, or REM. In this study, annotations from the first sleep specialist were used.

Following preprocessing procedures commonly used in automatic sleep staging [29,32,33], we first standardize the dataset labels. The R&K labels in Sleep-EDF-20 and Sleep-EDF-78 are mapped to the five AASM stages [5]. Stages 0, 1, and 2 are relabeled as W, N1, and N2, respectively. Stages 3 and 4 are merged into N3, REM remains unchanged, and MOVEMENT and UNKNOWN epochs are excluded. ISRUC-S3 already follows the AASM criteria and therefore requires no label processing. Because the high proportion of wake epochs in whole-night recordings may further exacerbate class imbalance, we retain only the interval from 30 min before sleep onset to 30 min after final awakening in Sleep-EDF-20 and Sleep-EDF-78. Table 1 summarizes the demographic characteristics and class distributions of the three datasets.

To meet the input requirements of the multimodal PSG framework, Fpz-Cz and Pz-Oz were selected as the EEG channels for Sleep-EDF-20 and Sleep-EDF-78, with horizontal EOG and submental EMG used as the EOG and EMG modalities, respectively. For ISRUC-S3, C3-A2 and C4-A1 were selected as the EEG channels, with ROC-A1 and Chin EMG used as the EOG and EMG modalities, respectively.

The selected signals were preprocessed according to the characteristics of each modality and dataset. A fourth-order Butterworth infinite impulse response band-pass filter was applied in zero-phase mode. In the Sleep-EDF Sleep Cassette recordings, EEG and EOG were sampled at 100 Hz and filtered at 0.2–40 Hz, whereas the available submental EMG was provided as a 1 Hz envelope and was not additionally filtered [38]. In ISRUC-S3, EEG, EOG, and Chin EMG were originally sampled at 200 Hz [40]. EEG and EOG were filtered at 0.2–40 Hz, while Chin EMG was filtered at 10–70 Hz to account for its relatively higher-frequency muscle activity. The 1 Hz Sleep-EDF EMG envelope was temporally aligned to 100 Hz during EDF loading in MNE-Python (version 1.9.0). The selected ISRUC-S3 signals were resampled from 200 to 100 Hz using the default fast Fourier transform-based resampling procedure in MNE-Python with anti-alias filtering, yielding a representation with a Nyquist frequency of 50 Hz, which means that frequency components above 50 Hz Nyquist limit are not represented in the final model input. The common 100 Hz representation ensured a consistent temporal resolution and input length across datasets and modalities. To reduce amplitude-scale differences across recordings, z-score normalization was performed independently for each PSG recording and each channel. The mean and standard deviation were calculated from all samples of the corresponding channel within that recording and were used only to normalize that channel. When multiple recordings were available for the same subject, each recording was normalized independently. For test recordings, these statistics were derived solely from the unlabeled input recording itself. No sleep stage labels or information from other recordings were used. The normalized continuous signals were finally divided into non-overlapping 30 s epochs, yielding 3000 samples per channel per epoch.

### 3.2. Experimental Protocol and Implementation Details

To assess performance on subjects not seen during training, we split each dataset by subject into *k* non-overlapping subsets. In each cross validation round, one subset serves as the test set, another as the validation set, and the remaining k−2 subsets constitute the training set. Thus, no subject appeared in more than one of the three sets within a round. After all *k* rounds, we combined predictions from the test sets to calculate the overall performance metrics. The evaluation uses 20-fold cross validation for Sleep-EDF-20 and 10-fold cross validation for Sleep-EDF-78 and ISRUC-S3. This setup tests the model on subjects not encountered during training while avoiding subject-level data leakage. The same subject splits were used in all subsequent comparison and ablation experiments.

Experiments were conducted on a workstation equipped with an NVIDIA GeForce RTX 2080 Ti GPU (NVIDIA Corporation, Santa Clara, CA, USA) using Python 3.10.19 and PyTorch 2.5.0. End-to-end training used a batch size of 128. The intra-modal, cross-modal, and inter-epoch attention modules each used eight attention heads, with a token embedding dimension of 128 and a dropout rate of 0.1. The feed-forward hidden dimension was 512. Adam was used for optimization with an initial learning rate of $5\times 10^{-4}$, $\beta_{1}=0.9$, $\beta_{2}=0.999$, and $\epsilon =1\times 10^{-9}$. An $L_{2}$ weight decay of $5\times 10^{-3}$ was applied to reduce overfitting. To mitigate class imbalance, the loss function used weighted cross-entropy with class weights of [1.0,2.0,1.0,2.0,2.0] for W, N1, N2, N3, and REM, respectively. Training was limited to 100 epochs, with early stopping after 20 consecutive epochs without improvement in validation accuracy, and the model with the highest validation accuracy was used for testing. Under the above hardware configuration, the average training time on Sleep-EDF-20 was approximately 1.3 h per fold. To further illustrate the training and convergence behavior, the training and validation loss and accuracy curves from a randomly selected fold of Sleep-EDF-20 are provided in Supplementary Figure S1.

During testing, inference is performed using a sliding window over each whole-night PSG recording. For epochs that can serve as the center of a complete context window, classification uses the final logits at the center position of the corresponding window. With the default sequence length of 9, the first and last 4 epochs cannot serve as window centers. To obtain complete whole-night hypnograms and evaluation metrics, predictions from overlapping windows [8,32] are aggregated for these boundary epochs. Specifically, predictions from all valid windows covering a boundary epoch are collected, and the corresponding final logits are converted to class probabilities by Softmax and averaged. The class with the highest mean probability is taken as the final prediction. This procedure is applied only during testing and does not affect model training. A sensitivity analysis comparing the default overlap-averaging fallback with boundary epoch exclusion and reflection padding is presented in Supplementary Table S1.

In addition, we report model complexity and computational inference efficiency. Measurements used a batch size of 1 with nine consecutive 30 s epochs sampled at 100 Hz. The model has 3.418 M trainable parameters and requires 397.10 million floating-point operations (MFLOPs). For a complete 9-epoch input sequence, the average model-execution latency is 44.7 ms, with peak GPU memory of approximately 95.61 MB. The reported latency refers to the forward computation performed after the complete input sequence is available. For offline whole-night PSG analysis, the model is applied sequentially to overlapping 9-epoch windows, with the overall computational cost increasing with recording duration. In practical biomedical monitoring systems, deployment-related timing can additionally be influenced by signal acquisition, processing hardware, data transmission, and the implementation platform [41].

### 3.3. Evaluation Metrics and Statistical Methods

To evaluate five-class sleep staging performance, we report accuracy (ACC), macro F1 score (MF1), Cohen’s κ, and the F1 score for each sleep stage [42]. Let *C* denote the number of classes, with C=5 corresponding to W, N1, N2, N3, and REM. For each stage, a one-vs-rest setting is used, with that stage treated as positive and all others as negative. True positives (TP) and true negatives (TN) are correctly classified positive and negative samples, respectively, whereas false positives (FP) and false negatives (FN) are negative samples classified as positive and positive samples classified as negative, respectively.

Accuracy measures the overall proportion of correctly classified samples:

(20) $$\mathrm{ACC}=\frac{\sum_{i=1}^{C}TP_{i}}{N},$$

where $TP_{i}$ is the number of correctly classified samples in class *i*, and *N* is the total number of test samples.

The F1 score is the harmonic mean of precision and recall. MF1 is the arithmetic mean of the class-wise F1 scores and measures overall classification performance across sleep stages. The corresponding formulas are:

(21) $$\begin{array}{cccc} {\mathrm{Prec}}_{i} & =\frac{TP_{i}}{TP_{i}+FP_{i}},\; & {\mathrm{Rec}}_{i} & =\frac{TP_{i}}{TP_{i}+FN_{i}},\\ F1_{i} & =\frac{2{\mathrm{Prec}}_{i}{\mathrm{Rec}}_{i}}{{\mathrm{Prec}}_{i}+{\mathrm{Rec}}_{i}},\; & \mathrm{MF}1 & =\frac{1}{C}\sum_{i=1}^{C}F1_{i}, \end{array}$$

where ${\mathrm{Prec}}_{i}$, ${\mathrm{Rec}}_{i}$, and $F1_{i}$ denote the precision, recall, and F1 score for class *i*, respectively.

Cohen’s κ measures the overall agreement between predicted and ground-truth labels beyond chance. It is computed by the formula:

(22) $$\kappa =\frac{P_{o}-P_{e}}{1-P_{e}},$$

where $P_{o}$ is the observed agreement between predicted and ground-truth labels and equals accuracy in this single-label multiclass task. $P_{e}$ is the agreement expected by chance from the marginal class distributions of the ground-truth and predicted labels:

(23) $$P_{e}=\sum_{i=1}^{C}p_{i}q_{i},$$

where $p_{i}$ and $q_{i}$ denote the proportions of class *i* in the ground-truth labels and predictions, respectively. A value of κ=1 indicates complete agreement, κ=0 indicates that observed agreement equals the chance agreement derived from the marginal class distributions, and κ<0 indicates agreement below chance.

To characterize variability across cross-validation partitions, the mean and 95% confidence intervals (CIs) of the fold-wise ACC, MF1, and Cohen’s κ were estimated using Student’s *t*-distribution with n−1 degrees of freedom, where *n* denotes the number of cross-validation folds. For configurations evaluated using identical subject splits, statistical differences in ACC, MF1, and κ were assessed using two-sided paired *t*-tests on the fold-wise results. When multiple related configurations were compared with the same reference within an experimental analysis, the resulting *p*-values were adjusted using the Holm procedure separately for ACC, MF1, and κ. A Holm-adjusted *p*-value below 0.05 was considered statistically significant. Exact raw and Holm-adjusted *p*-values for the ablation analyses are provided in Supplementary Table S2.

### 3.4. Compared Methods

To evaluate the automatic sleep staging performance of the proposed method, we compare it with representative single-epoch and multi-epoch context modeling methods. This categorization refers to the temporal context used for each prediction.

Single-epoch modeling methods take physiological signals from a single 30s epoch as input, extract sleep-related features within the epoch, and model relationships among signals when multichannel or multimodal inputs are used. Representative methods include SAN [43], MultiChannelSleepNet [23], MMASleepNet [44], MMTSleepNet [19], MixSleepNet [45], ADG-RANet [46], and SleepRWKVNet [33]. Multi-epoch context modeling methods take physiological signals from multiple consecutive epochs as input and model temporal relationships across adjacent epochs. Representative methods include DeepSleepNet [15], SalientSleepNet [22], AttnSleep [16], XSleepNet [31], MSSENet [47], SleepFC [17], Cross-Modal Transformer [32], SleePyCo [12], MFCR-SleepNet [14], and 3DSleepNet [48].

## 4. Results

### 4.1. Confusion Matrix Analysis

**Figure 6:**
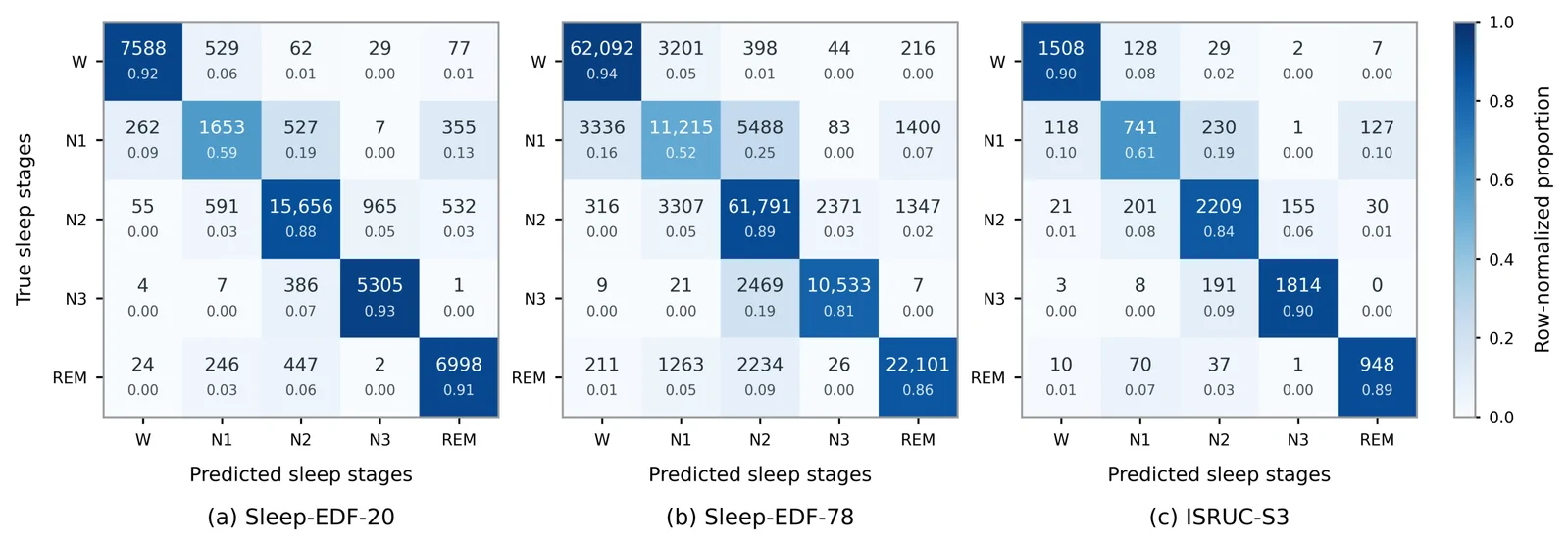
Confusion matrices of CSPT-SleepNet on the three evaluated datasets. In each cell, the upper value indicates the absolute number of epochs, and the lower value indicates the proportion normalized by the corresponding ground-truth class. The background color also represents the row-normalized proportion.

Figure 6 shows the confusion matrices of CSPT-SleepNet on the three datasets, obtained by pooling predictions from the test sets across all folds. All three matrices show similar patterns with clear diagonal dominance, indicating that CSPT-SleepNet distinguishes most sleep stages well. Recall exceeds 90% for W and remains above 80% for N2, N3, and REM on all datasets. In contrast, N1 has markedly lower recall than the other stages and remains the main recognition bottleneck.

Closer examination shows similar confusion patterns across the three datasets. Misclassified N1 samples are mainly assigned to W, N2, and REM, most often to N2. Thus, N1 errors are not confined to a single class, and the stronger confusion with N2 may reflect feature overlap between the two stages. Misclassification also occurs between N2 and N3, but the directions are asymmetric. Across all three datasets, no more than 6% of N2 samples are classified as N3, whereas N3 is more often classified as N2. Most of this confusion is due to N3 samples being classified as N2, with fewer errors in reverse. Error rates for the other class pairs are generally low, showing that misclassifications are concentrated in a few difficult stage pairs rather than broadly distributed across sleep stages.

Across the three datasets, CSPT-SleepNet mainly confuses N1 with W, N2, and REM, while some N3 epochs are misclassified as N2. N1 errors may be related to its transitional position between wakefulness and more stable sleep stages and to feature overlap with other stages [5,13]. Misclassification of N3 as N2 may reflect the mixed characteristics of epochs near N2–N3 transitions [7,13]. CSPT-SleepNet distinguishes most sleep stages well, with the remaining errors concentrated in stage pairs that are commonly difficult to separate in automatic sleep staging. Corresponding class-wise evaluation metrics based on the same pooled predictions are reported in Supplementary Table S3.

### 4.2. Overall Performance Comparison

Table 2 compares CSPT-SleepNet with representative sleep staging methods on Sleep-EDF-20, Sleep-EDF-78, and ISRUC-S3. The comparison methods may differ in channel or modality selection, preprocessing, input-context configuration, and evaluation protocol, and the results reported for these methods are taken from their corresponding original publications.

On Sleep-EDF-20, CSPT-SleepNet reports ACC, MF1, and κ values of 87.93%, 83.53%, and 0.836, respectively. Across the 20 cross-validation folds, the 95% CIs for the mean fold-wise ACC, MF1, and Cohen’s κ were 87.26–88.63% for ACC, 80.75–83.55% for MF1, and 0.822–0.842 for Cohen’s κ. Relative to the values reported for MMTSleepNet, which also uses EEG, EOG, and EMG modalities, the corresponding differences are 0.43 and 0.83 percentage points in ACC and MF1, respectively, and 0.006 in κ. At the class level, the reported results in Table 2 show favorable F1 performance for CSPT-SleepNet in W, N1, and REM, while the results for N2 and N3 are broadly comparable with those reported by the other methods. This stage-wise pattern suggests that the overall performance is not driven by improvement in a single class.

On Sleep-EDF-78, CSPT-SleepNet reports an ACC of 85.81%, an MF1 of 80.87%, and a κ of 0.804. Across the 10 cross-validation folds, the 95% CIs estimated from the fold-wise results were 85.04–86.51% for ACC, 78.97–82.03% for MF1, and 0.793–0.812 for Cohen’s κ. At the class level, the F1 scores of CSPT-SleepNet for N1 and REM are among the higher reported values summarized in Table 2, together with F1 scores of 94.1% for W and 87.3% for N2. In contrast, the N3 F1 score is 80.7%, lower than that reported by several comparison methods. Approximately 19% of N3 epochs are misclassified as N2, while misclassification to the other stages is relatively limited. This indicates that the lower N3 performance on Sleep-EDF-78 is mainly associated with a localized N2–N3 confusion rather than a broad loss of discriminability across stages. This pattern is consistent with the transition-related ambiguity discussed in Section 4.1. SAN and MSSENet report higher N3 F1 scores but lower N1 F1 scores, at 37.0% and 27.1%, respectively. For CSPT-SleepNet, although N3 is not among its strongest stage-wise results, the class-wise performance remains broadly balanced across multiple sleep stages, resulting in an MF1 of 80.87%.

On ISRUC-S3, CSPT-SleepNet reports an ACC of 84.06%, an MF1 of 82.86%, and a κ of 0.795. Across the 10 cross-validation folds, the 95% CIs estimated from the fold-wise results were 82.72–85.21% for ACC, 79.89–83.61% for MF1, and 0.772–0.804 for Cohen’s κ. Relative to the values reported for SleepRWKVNet, the corresponding numerical differences are 0.76 percentage points in ACC, 0.96 percentage points in MF1, and 0.005 in κ. At the class level, the reported results in Table 2 show relatively strong F1 values for CSPT-SleepNet in N1, N3, and REM, together with F1 scores of 83.2% for N2 and 90.5% for W. Taken together, this class-wise pattern indicates that the overall performance is not concentrated in a single sleep stage.

To further assess sensitivity to training randomness, we perform four independent runs with different random seeds under identical settings and report the results as mean ± standard deviation. On Sleep-EDF-20, the ACC, MF1, and κ are 87.91%±0.16%, 83.58%±0.21%, and 0.835±0.002, respectively. The corresponding results on Sleep-EDF-78 are 85.86%±0.10%, 80.92%±0.13%, and 0.804±0.001, while those on ISRUC-S3 are 84.13%±0.16%, 82.92%±0.26%, and 0.795±0.002. These mean values are close to those in Table 2, and the small standard deviations indicate stable performance across training initializations.

Overall, CSPT-SleepNet shows consistently strong overall performance across the three evaluated datasets, with ACC ranging from 84.06% to 87.93%, MF1 from 80.87% to 83.53%, and κ from 0.795 to 0.836. The 95% CIs characterize the variation in performance across the subject-independent cross-validation folds. Analysis across multiple random seeds further shows limited variation across different training initializations, supporting the optimization stability of the model. The corresponding fold-wise ACC, MF1, and Cohen’s κ values are provided in Supplementary Figure S2.

### 4.3. Sleep Hypnogram and Error Pattern Analysis

**Figure 7:**
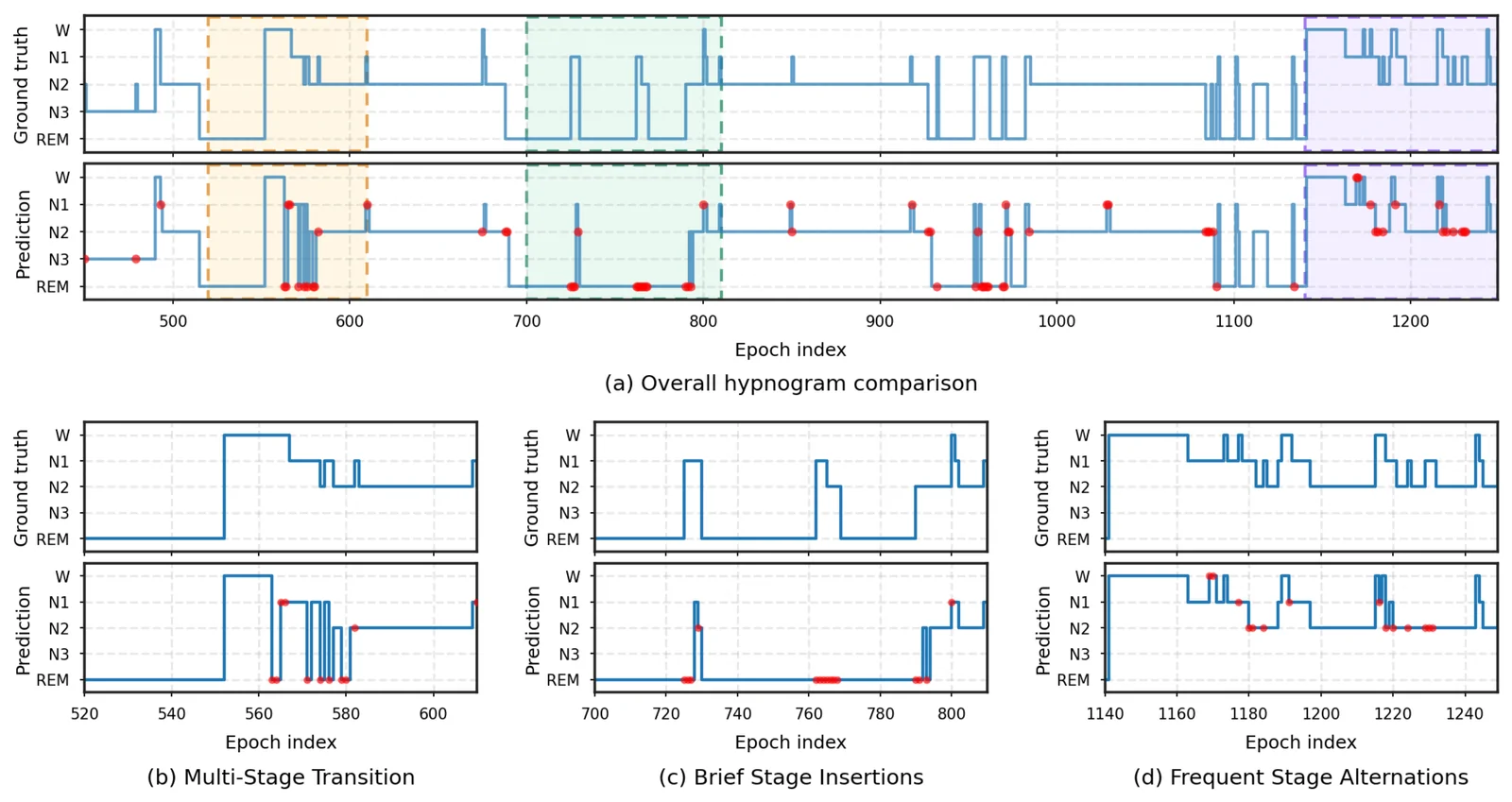
Sleep stage prediction sequence and examples of local errors produced by CSPT-SleepNet on Sleep-EDF-20. (**a**) Overall hypnogram comparison between the manually scored ground-truth and predicted sequences for epochs 450–1249 from the first night of Subject 19. (**b**) Multi-stage transition from REM through W and brief N1/N2 changes to N2. (**c**) Brief N1/N2 insertions within a longer REM interval. (**d**) Frequent alternation among W, N1, and N2. The orange, green, and purple shaded regions in (**a**) correspond to (**b**–**d**), respectively, and red dots indicate misclassified epochs.

Overall metrics and confusion matrices do not show when misclassifications occur in a continuous sleep sequence. We therefore analyzed the recording from the first night of Subject 19 from Sleep-EDF-20, which achieved an ACC of 88.14%, close to the overall dataset performance. Figure 7a shows the manually scored and predicted sleep stage sequences for an 800-epoch segment containing diverse stage transitions and local errors, and Figure 7b–d enlarge three representative regions involving multi-stage transitions, brief stage insertions, and frequent alternation between N1 and N2.

As shown in Figure 7a, the predicted sequence agrees with the manual scores across most intervals. Long W, N2, and REM segments are largely preserved, together with the overall stage progression. Misclassifications are concentrated around brief stage segments, rapid local transitions, and frequent alternation between adjacent stages rather than being evenly distributed across the sequence.

The three local regions illustrate different error patterns. In Figure 7b, the model correctly identifies the sustained REM segment at the beginning and the longer N2 interval at the end, while misclassifications are concentrated in the intermediate W segment and brief N1/N2 alternations. The overall progression from REM to N2 is retained, but local stage substitutions remain during rapid transitions. In Figure 7c, the dominant REM interval and subsequent stage changes are mostly identified correctly, but several brief N1 and N2 insertions are missed, with the model maintaining the REM prediction across these short intervals. Figure 7d shows that the transition from W to light sleep and the subsequent N1/N2 alternations are generally captured, although some brief N1 epochs are classified as W or N2 with slight shifts in local stage boundaries. This pattern is consistent with the N1 confusion observed in Section 4.1.

Overall, CSPT-SleepNet preserves longer stage segments and the main stage progression, while most errors occur in brief segments and local transition regions as continued prediction of the surrounding stage, local label substitution, or boundary shifts.

### 4.4. Comparative Evaluation of Center-Driven Sequence Supervision

To evaluate center-driven sequence supervision, we compare it with Equal-weight sequence supervision and Center-only sequence supervision on Sleep-EDF-20 in Table 3. To isolate the effect of supervision, TRC is removed from all three configurations, with base logits used directly for loss computation and center-epoch prediction. The three strategies differ only in how classification losses are assigned across positions in the final logit sequence, with all other settings unchanged. Equal-weight sequence supervision averages the classification losses of all epochs, and Center-only sequence supervision uses only the center-epoch loss. Center-driven sequence supervision follows the training objective and parameter settings defined in Section 2.7, with center-epoch classification as the primary objective and weaker auxiliary supervision applied to non-center epochs.

As shown in Table 3, Center-driven sequence supervision achieves the best ACC, MF1, and κ among the three configurations. The differences in these overall metrics were also statistically supported. After Holm correction, the comparisons with both Equal-weight and Center-only supervision remained significant for ACC, MF1, and κ, with adjusted *p*-values of 0.0476, 0.0331, and 0.0300, respectively, in both comparisons. The class-wise F1 scores further show how the MF1 gain is distributed across stages. F1 is higher for all five stages, with the largest gains over Equal-weight sequence supervision observed for N1 and REM, at 2.4 and 1.5 percentage points, respectively. N1 is one of the most difficult stages to classify, and its larger gain indicates a clearer improvement for this challenging class. Together with the improvement in REM and the gains observed for the other stages, this pattern suggests that the MF1 improvement is supported by improvements across classes and that incorporating the ground-truth labels of non-center epochs helps preserve stage-discriminative contextual information for center-epoch classification.

To further examine whether the performance improvement of center-driven sequence supervision was influenced by the overall loss scale, an additional control experiment was conducted using a normalized center-driven objective. Specifically, the original loss was normalized as $L_{\mathrm{CD},\mathrm{norm}}=\left(L_{\mathrm{center}}+0.3L_{\mathrm{nc}}\right)/1.3$, ensuring that the supervision coefficients summed to one, consistent with the equal-weight and center-only sequence supervision configurations. This normalization preserved the relative weighting between the center and non-center supervision terms, while the model architecture and other training settings remained unchanged. The normalized configuration achieved an ACC of 87.40%, an MF1 of 82.95%, and a κ of 0.829. Compared with the Equal-weight and Center-only sequence supervision configurations, it maintained higher performance across all three overall metrics. These differences remained statistically significant after Holm correction, with adjusted *p*-values of 0.0487 for ACC, 0.0434 for MF1, and 0.0378 for κ in both comparisons. These results support the effectiveness of asymmetric supervision weighting when the total supervision coefficients are held constant, indicating that the observed advantage cannot be explained solely by a larger overall loss scale. Detailed results and paired statistical comparisons are provided in Supplementary Section S6 (Table S4).

The sleep stage sequence analysis in Section 4.3 shows that some prediction errors are concentrated around stage transitions, whereas conventional classification metrics do not fully characterize such transition behavior. We therefore further evaluate post-transition stage recognition and transition behavior in the predicted sequence, as summarized in Table 4. Drawing on prior work on stage-boundary evaluation [14], a transition point is defined as a position where two adjacent epochs have different labels. Transition Onset Accuracy (TOA) is the proportion of ground-truth transitions for which the first epoch after the transition is correctly classified as the new stage. Pred./True Ratio is the ratio of predicted to ground-truth transition points. Missed Rate is the proportion of unmatched ground-truth transition points, whereas Spurious Rate is the proportion of unmatched predicted transition points. For these two metrics, ground-truth and predicted transition points are matched one-to-one within each PSG recording using a tolerance of ±2 epochs (±60 s), and each point is matched at most once. The robustness of the matching tolerance was further evaluated using ±1 and ±3 epochs, with the results provided in Supplementary Table S5.

Equal-weight sequence supervision achieves the Pred./True Ratio closest to 1 and the lowest Spurious Rate, but yields the lowest TOA and the highest Missed Rate. Similar predicted and true transition counts do not necessarily imply close temporal alignment, because missed true transitions may be offset in count by additional predictions at other positions. Center-only sequence supervision reduces the Missed Rate to 33.40%, but increases the Pred./True Ratio to 1.310 and the Spurious Rate to 49.16%, indicating that fewer ground-truth transitions are missed at the cost of more additional predicted transitions. Compared with Equal-weight sequence supervision, Center-driven sequence supervision increases TOA by 3.35 percentage points, the highest of the three configurations. Its Pred./True Ratio rises from 1.033 to 1.145, while the Spurious Rate increases by only 0.76 percentage points and the Missed Rate decreases by 5.51 percentage points. This configuration thus improves post-transition recognition and reduces missed true transitions with only a small increase in spurious transitions. Compared with Center-only sequence supervision, fewer transitions are predicted, although the Missed Rate is 2.53 percentage points higher. Overall, Center-driven sequence supervision provides a better balance between transition recognition, transition counts, and missed and spurious transitions.

Considering both classification and stage transition results, Center-driven sequence supervision achieves the best classification performance among the three configurations and shows more balanced transition behavior.

### 4.5. Ablation Study of Model Components and Multimodal Fusion

To assess the contribution of key model components and multimodal fusion design, seven ablation variants were evaluated on Sleep-EDF-20 in Table 5. The first three variants examine the main components of MPTE. w/o Hierarchical Pyramid Pathway retains the initial downsampling stem but replaces the hierarchical pyramid pathway with parallel multi-scale convolutional neural networks (CNNs). w/o Learnable Inter-Level Downsampling retains the hierarchical pyramid structure while replacing learnable inter-level downsampling with fixed max pooling. w/o Fine-Grained Supplementary Branch removes the corresponding component. For multimodal fusion, Early Fusion concatenates EEG, EOG, and EMG before feature extraction, Late Representation Fusion concatenates independently encoded modality representations followed by linear projection, and Attention-Based Fusion (w/o JMR) retains cross-modal attention while removing JMR. The w/o TRC configuration, identical to Center-driven sequence supervision in Table 3, removes the residual logit correction branch and the associated auxiliary supervision on the base logits. Other settings were kept unchanged unless required by the corresponding structural modification.

The largest performance drop occurs after removing the hierarchical pyramid pathway, with ACC and MF1 decreasing by 0.97 and 1.14 percentage points, respectively, and κ by 0.014. The differences remain statistically significant after Holm correction for all three overall metrics, showing that the degradation is reflected in overall accuracy, class-balanced performance, and prediction agreement. Because this variant retains multiple receptive fields but removes progressive cross-scale propagation and integration, the degradation suggests that hierarchical cross-scale integration plays an important role in effective multi-scale intra-epoch representation.

Compared with the full model, replacing learnable inter-level downsampling with fixed max pooling reduces ACC and MF1 by 0.48 and 0.43 percentage points, respectively, whereas removing the fine-grained supplementary branch leads to reductions of 0.61 and 0.67 percentage points. Both degradations are smaller than that from removing the hierarchical pyramid pathway because the pyramid structure and feature propagation across levels are still retained. The statistical results further distinguish the effects of these two modifications. After Holm correction, statistical support for removing the fine-grained supplementary branch was retained for MF1 and κ, whereas the evidence was more limited for ACC and for the fixed max-pooling variant across the three overall metrics. This difference is consistent with the roles of the two components in MPTE. Fixed max pooling changes only the inter-level downsampling operation while retaining the hierarchical pathway, whereas removing the fine-grained supplementary branch eliminates a high-resolution feature route designed to preserve local information that may be weakened by progressive downsampling. Across these three MPTE ablations, N1 and REM generally show larger F1 losses than the other stages, which may reflect their greater dependence on fine local patterns and information represented across multiple temporal scales.

The multimodal fusion comparison shows a consistent improvement from input-level fusion to modality-specific representation learning and explicit cross-modal interaction. Compared with Early Fusion, the Full model improves ACC and MF1 by 0.84 and 0.87 percentage points, respectively, while Late Representation Fusion and Attention-Based Fusion yield intermediate performance, suggesting that preserving modality-specific representations and explicitly modeling cross-modal dependencies are both beneficial within the current framework. JMR provides a further refinement over Attention-Based Fusion, increasing ACC and MF1 by 0.40 and 0.45 percentage points, respectively. At the class level, the largest improvement from Early Fusion to the Full model is observed for N1, followed by REM, whereas the changes for W, N2, and N3 are smaller. Taken together, these results suggest that preserving modality-specific representations and explicitly modeling cross-modal dependencies are beneficial within the current framework, with JMR providing a further refinement. For the three fusion variants, statistical support after Holm correction was retained for κ, whereas the evidence for ACC and MF1 was comparatively weaker.

Removing TRC decreases ACC by 0.46 percentage points and MF1 by 0.45 percentage points. The differences were statistically supported across all three overall metrics. This modest degradation is consistent with the role of TRC as a refinement component that learns supplementary corrections from the encoded inter-epoch context representation and base logit sequence to refine the final predictions.

Overall, the ablation results support the roles of hierarchical multi-scale representation, structured multimodal fusion, and output refinement in CSPT-SleepNet, with MPTE providing the main representation basis and JMR and TRC serving as subsequent refinement components. The corresponding parameter counts and MFLOPs of these variants are provided in Supplementary Table S6.

### 4.6. Modality Contribution Analysis

To assess the effects of different physiological modalities on sleep staging, we evaluated four input configurations on Sleep-EDF-20 with EEG as the base modality, as reported in Table 6. For each configuration, only the selected modality branches were instantiated, with JMR and cross-modal interaction adjusted accordingly, and all other model and training settings remained unchanged.

With EEG alone, the model achieved an ACC of 86.01% and an MF1 of 81.22%, showing that EEG provides strong baseline discriminative information. Adding either EOG or EMG improved overall performance, with EEG+EOG yielding higher ACC, MF1, and κ than EEG+EMG. Thus, EOG provided a larger overall gain under the current model and data settings. For both auxiliary modalities, the improvements were mainly observed for N1 and REM, suggesting that EOG and EMG provide additional discriminative information beyond EEG. This pattern is consistent with the known physiological roles of eye movements and muscle tone [9,19,33].

The EEG+EOG+EMG input achieved the highest overall performance. In terms of class-wise F1, the largest improvement over EEG+EOG was observed for N1, whose F1 score increased by 3.4 percentage points, while W, N2, N3, and REM also improved. The improvement may partly arise from the additional muscle tone cues provided by EMG, complementing the brain activity and eye movement information captured by EEG and EOG. For N3, the F1 score remained within a narrow range of 87.9–88.3% across the four modality configurations, with 88.1% for EEG alone and 88.3% for the full three-modality input. This limited variation suggests that N3 performance changes little with modality combination under the current model and data settings, in contrast to the clearer gains observed for N1 and REM.

Overall, the modality ablation results show that EEG provides the main discriminative basis, while EOG and EMG contribute complementary information, particularly for N1 and REM. The Holm-adjusted analysis further supported the ACC and κ differences between the full multimodal configuration and all reduced-modality settings, while statistical support for the MF1 difference was retained for EEG alone. These trends are also reflected in the modality weight statistics across sleep stages provided in Supplementary Figure S3. The computational complexity of the different modality configurations is also reported in Supplementary Table S6.

### 4.7. Parameter Sensitivity Analysis

This section examines the effects of input context length *S* and non-center auxiliary supervision weight $\lambda_{\mathrm{nc}}$ on classification performance on the Sleep-EDF-20 dataset. For the context length experiment, we evaluated S=3, 5, 7, 9, and 11 to provide the same number of context epochs on both sides of the center epoch, and compared these settings with an independent epoch-level reference model that classifies each epoch directly from its cross-modal representation. For auxiliary supervision, *S* was fixed at 9 and only $\lambda_{\mathrm{nc}}$ was varied from 0.0 to 0.5 in increments of 0.1. At $\lambda_{\mathrm{nc}}=0$, only auxiliary supervision on the non-center final logits was removed, while TRC and auxiliary supervision on the base logits were retained.

**Figure 8:**
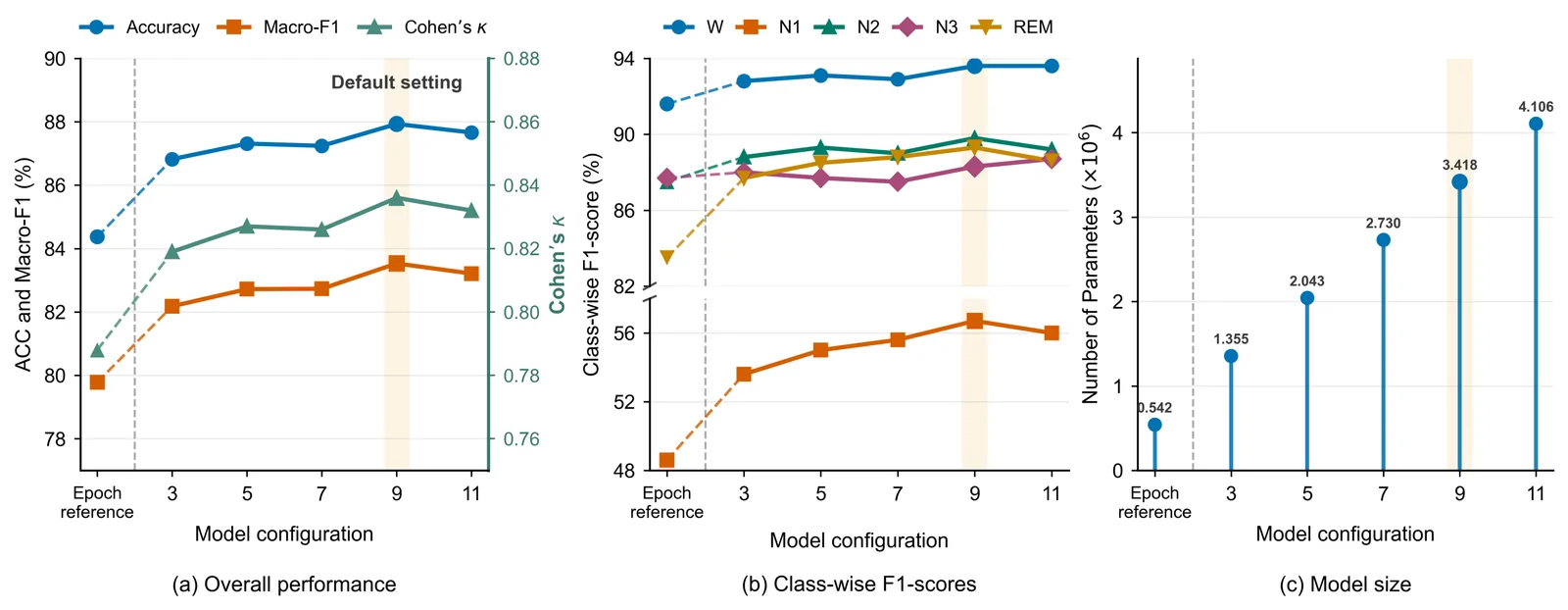
Effects of input context length on classification performance and model size on Sleep-EDF-20. (**a**) Overall classification metrics, (**b**) class-wise F1 scores, and (**c**) number of trainable parameters. The shaded region marks the S=9 setting used in this study. The vertical gray dashed lines separate the independent epoch-level reference from the multi-epoch configurations, while the colored dashed segments in (**a**,**b**) visually connect the reference results to those of the three-epoch configuration.

As shown in Figure 8a,b, S=3 improves ACC and MF1 over the independent epoch-level reference. The F1 scores for N1 and REM increase by 5.0 and 4.2 percentage points, respectively. These gains suggest that, beyond epoch-level multimodal modeling, introducing adjacent epochs and inter-epoch context modeling provides useful local stage context for center-epoch classification, particularly for N1 and REM, whose characteristics can overlap with those of adjacent stages. With consecutive epochs as input, performance does not improve monotonically as *S* increases. The results for S=5 and S=7 are similar, whereas S=9 achieves the highest ACC, MF1, and κ, followed by declines in all three metrics at S=11 despite a slight increase in N3 F1. This result suggests that more distant context benefits different stages unevenly and that a class-specific gain does not necessarily improve overall performance. With 30s epochs, S=9 spans 4.5 min and provides 2 min of context on each side of the center epoch, offering a local temporal range for modeling stage persistence and transition-related changes. As shown in Figure 8c, the parameter count increases approximately linearly with *S*, while overall performance no longer improves beyond S=9. Among the tested settings, S=9 achieves the highest overall classification performance. S=5 retains most of the performance with 2.043 M parameters and offers a more compact option when model size is prioritized.

**Figure 9:**
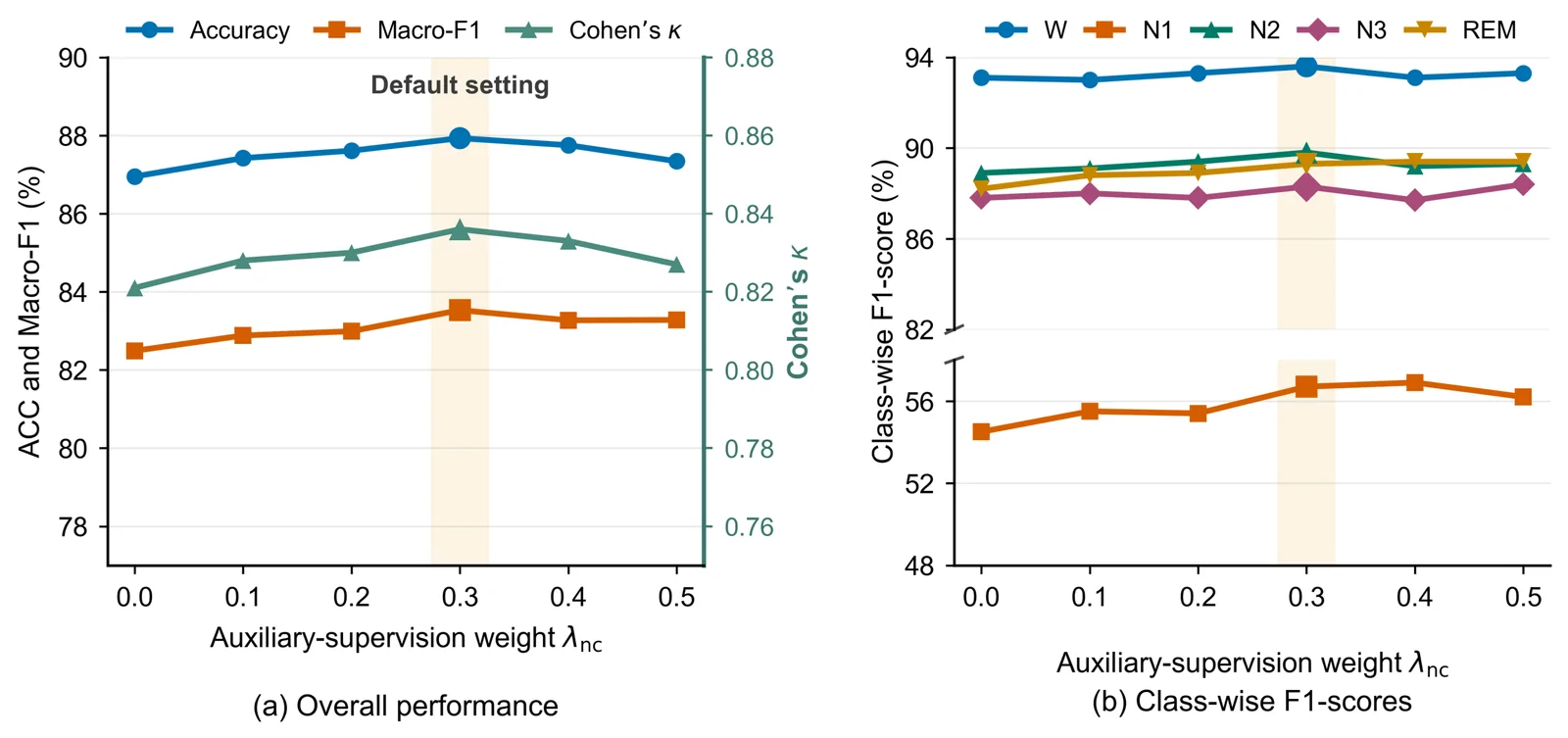
Classification results for different non-center auxiliary supervision weights on Sleep-EDF-20. (**a**) Overall classification metrics and (**b**) class-wise F1 scores. The shaded region marks the $\lambda_{\mathrm{nc}}=0.3$ setting used in this study.

As shown in Figure 9, ACC, MF1, and κ generally improve as $\lambda_{\mathrm{nc}}$ increases from 0 to 0.3, indicating that auxiliary supervision on the non-center epochs helps neighboring representations retain stage-discriminative information that is useful for center-epoch classification. The improvement, however, does not continue when $\lambda_{\mathrm{nc}}$ is increased from 0.3 to 0.5. The overall metrics remain largely stable or decrease slightly, while only small stage-specific variations are observed in the class-wise F1 scores. Taken together, these results suggest that $\lambda_{\mathrm{nc}}$ regulates the relative influence of non-center supervision during optimization. A moderate weight allows stage information from neighboring epochs to contribute to the center prediction without placing excessive emphasis on the non-center objectives relative to the center-epoch objective. Among the evaluated settings, $\lambda_{\mathrm{nc}}=0.3$ achieves the best overall performance.

### 4.8. Hierarchical Context and Multimodal Interaction Analysis

This section further examines hierarchical information allocation in individual predictions across context positions, input modalities, and local intra-modal feature tokens. For an input window, $\alpha_{c,j}$ denotes the inter-epoch attention from the center position *c* to position *j*, $w_{j,m}$ denotes the relative weight of modality *m* at position *j*, and $u_{j,m,n}$ denotes the attention from the modality CLS token to local feature token *n*. All attention weights are obtained by averaging the corresponding multi-head attention weights over the attention heads. $\alpha_{c,j}$, $w_{j,m}$, and $u_{j,m,n}$ are normalized across input positions, modalities at each position, and local tokens within each modality, respectively. Additional examples across all five sleep stages are provided in Supplementary Figure S4, illustrating variations in inter-epoch attention and position-specific modality weights across different stage contexts.

The intra-modal hierarchical relevance score is defined as

(24) $$\rho_{j,m,n}=\alpha_{c,j}w_{j,m}u_{j,m,n}.$$

**Figure 10:**
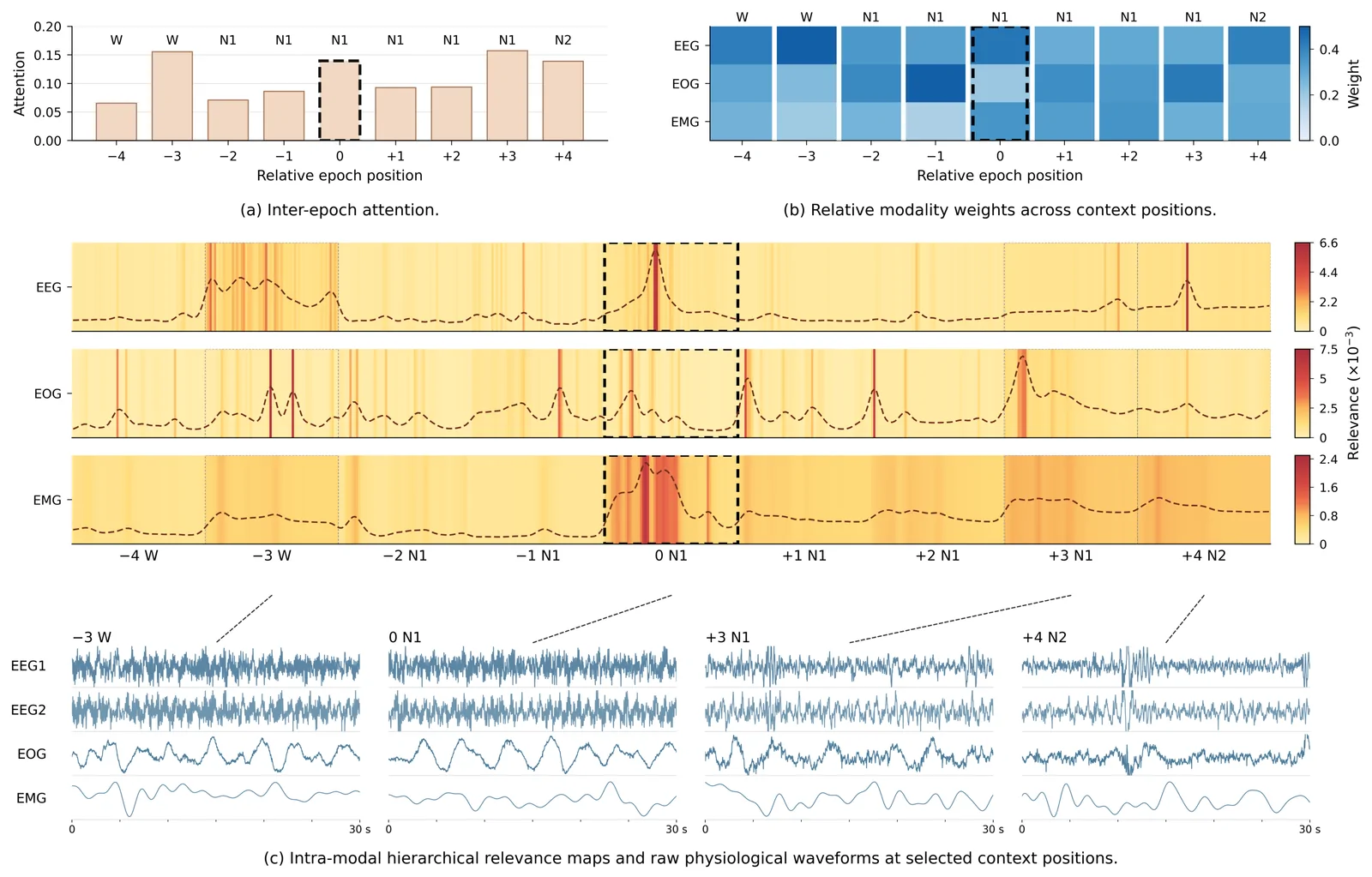
Example of hierarchical information allocation for N1 classification at the center position in a W–N1–N2 transition context. (**a**) Inter-epoch attention, (**b**) relative modality weights at each context position, and (**c**) intra-modal hierarchical relevance maps and raw physiological waveforms for four high-attention positions. Separate color scales are used for the three modalities in (**c**) to visualize within-modality relevance patterns. Black dashed boxes indicate the center epoch in panels (**a**–**c**).

This score combines center-epoch attention across input positions, modality information allocation at that position, and local token responses within that modality, linking the information distributions across the three levels. Figure 10 illustrates a correctly classified example with N1 at the center in a W–N1–N2 context. Separate color scales are used for each modality, and color intensity is compared only within the same modality.

As shown in Figure 10a, high inter-epoch attention appears at the center N1 epoch, the preceding W epoch at relative position −3, the N1 epoch at +3, and the N2 epoch at +4. These positions include both same-stage N1 epochs and neighboring W and N2 epochs, suggesting that the inter-epoch attention is distributed over both stage persistence and adjacent transition context rather than being concentrated only on the center epoch. Figure 10b shows that modality weights within each position are not determined solely by the stage label. Although positions −1, 0, and +3 are all N1, their modality weights differ. EOG dominates at −1, EEG and EMG receive relatively high weights at the center, and at +3, EOG receives a higher weight than EEG and EMG, which have similar weights. This variation suggests that modality weights are not fixed for a given sleep stage and can vary across positions within the same stage.

Figure 10c further incorporates intra-modal token attention to show hierarchical relevance distribution across context positions. For the center N1 epoch, several EEG and EMG feature tokens show relatively high relevance. Its raw EEG generally exhibits low-amplitude mixed-frequency activity without clear sleep spindles or K-complexes, consistent with common physiological characteristics of N1 [5,9]. The epoch at relative position +3 is also N1, but EOG shows a pronounced local high-relevance region at this position. Its raw EOG shows pronounced local fluctuations, suggesting possible eye movement activity associated with N1 in this region [5]. At relative position +4, the N2 epoch is dominated by EEG modality weight, and its raw EEG contains a prominent transient with some morphological similarity to a K-complex [5]. This example shows hierarchical information allocation across inter-epoch attention, modality weights within each position, and local intra-modal responses. For this example, the attention and relevance distributions extend across the local W–N1–N2 stage context, with different modality weights and local token responses across positions.

To further examine whether the highlighted regions were associated with changes in the model output rather than only reflecting the internal distribution of attention coefficients, we conducted an occlusion experiment on this representative sample. For each of the nine context positions and three modalities, the local region with the highest relevance was compared with a low-relevance region of equal length from the same position and modality by setting the corresponding normalized input segment to zero. With a 1.5-s occlusion window, the high-relevance regions produced larger absolute changes in the center-stage probability in 25 of the 27 comparisons across epochs and modalities. The same pattern was observed with 1.0-s and 2.0-s windows in 26/27 and 24/27 comparisons, respectively. In addition, cumulative occlusion of the Top-1, Top-3, and Top-5 high-relevance regions produced larger absolute probability changes than the corresponding matched low-relevance regions under all three window lengths. These results show that, in this example, the center-stage output is generally more sensitive to perturbations of regions with higher hierarchical relevance. Detailed results of the occlusion experiment are provided in Supplementary Table S7.

Overall, the analysis shows that information allocation varies across context positions, modalities within each position, and local intra-modal features. The occlusion experiment further shows that regions with higher hierarchical relevance are generally associated with greater changes in the model output for this example. Together, these results illustrate a hierarchical information allocation pattern from inter-epoch context to modality and local features.

## 5. Discussion

### 5.1. Effectiveness of Center-Driven Sequence Supervision

Previous studies have shown that adjacent epochs provide information on stage persistence and transitions, improving the temporal context representation of the target epoch [26,29,31,49]. We further examined the effects of different sequence supervision strategies on center-epoch classification using consecutive epochs as input. With the network architecture and evaluation procedure unchanged, Center-driven sequence supervision achieved the best overall classification performance and TOA among the three strategies. Compared with Equal-weight sequence supervision, this strategy reduced the Missed Rate with only a slight increase in the Spurious Rate. Compared with Center-only sequence supervision, it showed a slightly higher Missed Rate but a clearly lower Spurious Rate, with the Pred./True Ratio closer to 1. These results suggest that center-driven sequence supervision improves not only overall classification performance but also the balance of transition prediction behavior jointly characterized by TOA, Pred./True Ratio, Missed Rate, and Spurious Rate.

Supervising only the center epoch leaves the representations of the remaining epochs without direct stage supervision. When all epochs are supervised equally, the optimization priority of the center classification objective is reduced. Center-driven sequence supervision addresses this trade-off by assigning a smaller loss weight to non-center epochs. It therefore supplies direct stage constraints to non-center epoch representations while preserving the dominant role of the center objective, which is better aligned with the center-epoch prediction task. Equal-weight and Center-driven sequence supervision both use the ground-truth labels of all epochs within the input sequence. Their difference lies only in the relative contribution assigned to center and non-center epochs during optimization. The better performance of Center-driven sequence supervision therefore suggests that the weighting of these supervised objectives, rather than the introduction of additional supervised targets alone, contributes to the observed improvement. The additional matched-scale control further supports this interpretation, as the normalized Center-driven configuration retained statistically significant improvements over both alternative supervision strategies after controlling for the total supervision coefficient. The larger F1 gain for N1 further suggests that incorporating the ground-truth labels of non-center epochs provides useful stage-informed contextual support for this challenging stage.

The sensitivity analysis of the non-center auxiliary supervision weight in Section 4.7 further supports this balance. Within the candidate range examined, moderate auxiliary supervision yielded the best overall results, whereas increasing $\lambda_{\mathrm{nc}}$ further brought no additional improvement. These results suggest that non-center supervision is most useful when it provides stage-discriminative constraints while remaining secondary to the center-epoch objective. Together, the comparison of supervision strategies and sensitivity analysis show that center-driven sequence supervision combines a dominant center-epoch objective with weaker non-center auxiliary supervision, allowing neighboring label information to contribute to center-epoch prediction without giving all sequence positions equal emphasis during optimization.

### 5.2. Architectural Design and Hierarchical Information Integration

Component ablation results show that MPTE not only extracts features at different receptive fields but also links short-term local waveforms with rhythmic features at longer temporal scales through hierarchical propagation. The hierarchical pyramid pathway progressively increases the receptive field. Learnable inter-level downsampling preserves informative features during scale changes, and the fine-grained supplementary branch reduces the loss of local details after successive compression. Compared with independent parallel multi-scale branches, this hierarchical design yields more coherently organized multi-scale representations across levels and facilitates subsequent relationship modeling in a unified token space.

The modality contribution analysis shows that EEG provides the strongest single-modality basis under the current experimental setting, while adding EOG and EMG further improves classification performance. The fusion-strategy comparison provides a complementary view of how these multimodal representations are combined. Direct input-level fusion yields lower overall performance than retaining separate modality-specific encoders, while replacing simple representation concatenation with cross-modal attention provides a further improvement. JMR produces an additional gain when applied before cross-modal attention. The benefit of joint multimodal modeling therefore appears to arise not only from the inclusion of multiple information sources, but also from how their representations are organized and interacted within the fusion process.

Inter-epoch modeling encodes the cross-modal epoch tokens at all positions in temporal order, allowing the center epoch to use stage persistence and transition information from neighboring epochs. Compared with independent epoch modeling, adding consecutive epoch context improves the overall metrics and the F1 scores for N1 and REM, suggesting that neighboring epochs aid center-epoch classification. The results for different sequence lengths further show that moderately extending the context improves performance, whereas more distant epochs provide no additional benefit. This agrees with previous findings that adjacent epoch context helps discriminate difficult stages [13,14]. Temporal Residual Correction further refines the classification outputs.

Detailed parameter counts and MFLOPs for the component, fusion, modality, and epoch level configurations are provided in Supplementary Table S6. Overall, computational complexity and classification performance do not change proportionally across different configurations. Replacing the hierarchical pyramid pathway with parallel multi-scale CNNs reduces performance despite higher computational cost, suggesting that the benefit of the hierarchical design does not simply arise from greater computation. Simpler fusion strategies, fewer input modalities, and epoch level modeling reduce computational cost but are also accompanied by lower classification performance. Thus, the full configuration favors richer multimodal and temporal modeling for offline PSG analysis, while simplified configurations provide alternatives when computational resources or signal availability are limited.

Hierarchical information allocation also varies across context positions, input modalities, and local intra-modal features for different inputs, consistent with the model structure for hierarchical modeling of inter-epoch context and multimodal information. CSPT-SleepNet thus progressively integrates multi-scale physiological features, complementary multimodal cues, and local stage context through multi-scale representation learning, intra-modal and cross-modal interaction, and inter-epoch modeling to form hierarchical representations for center-epoch classification. Although inter-epoch context provides useful information about local stage persistence and transitions, its benefit is still limited when a transition occurs within a fixed 30s epoch, where characteristics of adjacent stages may coexist under a single stage label. This may partly account for the remaining boundary shifts and confusion between adjacent stages observed in overnight predictions.

### 5.3. Applicability and Limitations

The proposed method uses synchronized EEG, EOG, and EMG recordings in a multimodal PSG setting. It combines complementary information from brain activity, eye movements, and muscle tone with multi-scale features and contextual information from neighboring epochs to classify the center epoch. Compared with reduced-channel systems developed for easier acquisition and long-term monitoring, CSPT-SleepNet is mainly applicable to laboratory PSG analysis and other settings where synchronized multimodal recordings are available. This study has several limitations:

(1) The current model adopts relatively fixed settings in terms of structural configuration and training parameters. On the one hand, the model uses a fixed symmetric context window. Epoch encoders and classification heads at different sequence positions have the same structure but separate parameters. This makes the model dependent on a preset sequence length, while also increasing parameter count and computation. In the current fixed-window design, this position-specific parameterization provides relative-position information, while parameter sharing with explicit positional encoding may offer a more flexible and efficient alternative. On the other hand, the relevant weighting coefficients used during training are predefined and remain fixed, without adaptive adjustment according to the data distribution or model uncertainty. The auxiliary supervision weight also warrants further evaluation across different data settings. Future work will explore variable length context modeling, parameter sharing across sequence positions, and lightweight encoding strategies to reduce model complexity, while further investigating methods that dynamically adjust the relevant weights according to data characteristics and training behavior, thereby improving adaptability and computational efficiency across different data settings.

(2) The current model uses a symmetric temporal window composed of consecutive multimodal epochs, which allows the contextual information before and after the center epoch to be fully utilized in offline PSG analysis. However, its dependence on future context limits its applicability to sleep staging scenarios that require low latency or online prediction. The current normalization strategy is also designed for offline recording-level analysis and would require adaptation for causal or online applications. The current experiments are mainly conducted under conditions where complete temporal context is available, and the effects of different temporal context constraints and hardware platforms on model performance and efficiency have not yet been systematically examined. Future work will investigate causal or limited look ahead temporal modeling and evaluate the trade off among performance, computational cost, and latency under different context settings and deployment platforms, with the aim of improving the applicability of the model to practical sleep monitoring.

(3) The current model assumes complete multimodal input with adequate signal quality. During PSG acquisition, however, electrode detachment, motion artifacts, and other recording problems may leave one or more signals missing or unreliable. Future work will investigate higher-rate or modality-specific EMG processing, together with compensation for missing modalities and signal-quality-aware fusion into the model and evaluate model performance under severe artifacts and systematic channel degradation to improve robustness to missing or unreliable signals. For settings with limited signal availability, reduced-channel configurations will also be examined for wearable monitoring.

(4) The current analysis of hierarchical information allocation combines attention-based visualization with occlusion analysis to examine hierarchical information allocation and the sensitivity of model output to high-relevance regions. However, the relationship between highly relevant local regions and specific sleep-related physiological events has not yet been systematically established. Future work will combine prototype learning, physiological event annotations, and attribution methods guided by physiological priors to further investigate this relationship.

(5) Each dataset was evaluated separately using a subject-independent protocol. The results therefore reflect within-dataset generalization to unseen subjects, and cross-dataset transfer was not examined. The relatively limited cohort sizes, particularly the 10-subject ISRUC-S3 dataset, also constrain the extent to which the current findings can be generalized to broader clinical populations. The datasets consist of healthy subjects, and the model has not been validated in patients with specific sleep disorders, such as sleep apnea or insomnia. Future work will include cross-dataset testing and external validation using data from other medical centers and clinical cohorts with specific sleep disorders. Domain generalization, self-supervised pretraining, and transfer learning will also be explored to reduce performance loss under distribution differences.

## 6. Conclusions

We propose CSPT-SleepNet, an automatic sleep staging framework that integrates center-driven sequence supervision with multimodal pyramid token encoding. The framework takes consecutive epochs as input and predicts the sleep stage of the center epoch. A Multimodal Pyramid Token Encoder extracts multi-scale physiological features from EEG, EOG, and EMG, followed by sequential modeling of intra-modal token relationships, complementary cross-modal information, and inter-epoch temporal relationships. During training, the center epoch receives primary classification supervision, whereas non-center epochs receive weaker auxiliary supervision based on their respective ground-truth labels. This encourages non-center epoch representations to retain stage-discriminative information and provide stage-informed contextual support for center-epoch classification.

Subject-independent evaluations on Sleep-EDF-20, Sleep-EDF-78, and ISRUC-S3 showed consistent overall classification performance across the three datasets. Comparisons of supervision strategies and MPTE component ablations supported the effectiveness of center-driven sequence supervision and MPTE, respectively, while additional component ablations provided further evidence for the roles of the remaining key modules. Center-driven sequence supervision also improved overall classification performance while yielding more balanced transition predictions. These results suggest that center-driven sequence supervision can exploit non-center epoch labels while preserving the primary role of center-epoch classification, and MPTE organizes and integrates multi-scale sleep-related features from multimodal PSG. Subsequent modeling further integrates multimodal features and inter-epoch contextual relationships. Future work will explore variable-length context modeling and parameter sharing, conduct cross-dataset testing and independent external validation, and assess robustness under missing modalities and low-quality signals.

## Figures and Tables

**Table 1:** Dataset demographics, statistics, and class distributions.

Dataset	Subjects	Age Range (Years)	Sex (M/F)	Avg. Epochs/ Subject	Original Sampling Rate	Class Distribution					Total Epochs
						W	N1	N2	N3	REM	
Sleep-EDF-20	20	25–34	10/10	2115	EEG/EOG: 100 Hz EMG env.: 1 Hz	8285 (19.58%)	2804 (6.63%)	17,799 (42.07%)	5703 (13.48%)	7717 (18.24%)	42,308
Sleep-EDF-78	78	25–101	37/41	2506		65,951 (33.74%)	21,522 (11.01%)	69,132 (35.37%)	13,039 (6.67%)	25,835 (13.22%)	195,479
ISRUC-S3	10	30–58	9/1	859	200 Hz	1674 (19.49%)	1217 (14.17%)	2616 (30.46%)	2016 (23.47%)	1066 (12.41%)	8589

*Note:* M/F denotes the number of male/female subjects; EMG env. denotes the submental EMG envelope signal provided in the Sleep-EDF Sleep Cassette recordings.

**Table 2:** Comparison of reported performance with representative sleep staging methods.

Dataset	Method	Input Epochs	Input Modalities	Overall Metrics			F1 Score for Each Class (%)				
				ACC (%)	MF1 (%)	κ	W	N1	N2	N3	REM
Sleep-EDF-20	DeepSleepNet	25	EEG	82.0	76.9	0.760	84.7	46.6	85.9	84.8	82.4
	SalientSleepNet	20	EEG, EOG	87.5	83.0	-	92.3	56.2	89.9	87.2	89.2
	AttnSleep	3	EEG	85.6	80.9	0.800	90.3	47.9	89.8	89.0	85.0
	XSleepNet	20	EEG, EOG	86.4	80.9	0.813	91.5	51.1	87.5	86.9	87.4
	MultiChannelSleepNet	1	EEG, EOG	87.2	81.2	0.82	92.8	49.1	90.0	89.3	84.8
	MSSENet	3	EEG	86.9	80.9	0.822	93.4	50.2	90.1	86.1	84.6
	SleepFC	10	EEG	86.8	81.5	0.818	90.8	53.0	89.6	87.0	87.2
	MMTSleepNet	1	EEG, EOG, EMG	87.5	82.7	0.830	92.6	55.4	90.0	90.0	85.7
	SleepRWKVNet	1	EEG, EOG, EMG	87.7	82.6	0.830	93.1	53.1	**90.3**	**90.3**	86.4
	CSPT-SleepNet (Ours)	9	EEG, EOG, EMG	**87.93**	**83.53**	**0.836**	**93.6**	**56.7**	89.8	88.3	**89.3**
Sleep-EDF-78	AttnSleep	3	EEG	82.9	78.1	0.770	92.6	47.4	85.5	83.7	81.5
	SAN	1	EEG	85.8	77.6	**0.854**	**94.5**	37.0	84.5	**96.8**	84.1
	MSSENet	3	EEG	84.3	74.4	0.783	91.7	27.1	**88.0**	87.5	77.8
	SleepFC	10	EEG	84.2	78.8	0.781	93.2	50.3	86.2	81.4	83.0
	Cross-Modal Transformer	15	EEG, EOG	84.3	79.4	0.785	93.5	54.3	85.5	77.0	86.7
	SleePyCo	10	EEG	84.6	79.0	0.787	93.5	50.4	86.5	80.5	84.2
	MFCR-SleepNet	10	EEG	**85.9**	79.7	0.789	93.7	55.1	86.9	86.0	86.7
	SleepRWKVNet	1	EEG, EOG, EMG	83.3	77.9	0.770	93.8	51.2	85.7	81.9	77.1
	CSPT-SleepNet (Ours)	9	EEG, EOG, EMG	85.81	**80.87**	0.804	94.1	**55.3**	87.3	80.7	**86.8**
ISRUC-S3	MMASleepNet	1	EEG, EOG, EMG	81.92	80.64	0.768	88.9	59.6	82.0	87.0	86.9
	3DSleepNet	Multiple	EEG, EOG, EMG	83.2	81.4	0.783	89.6	59.6	**83.2**	90.9	83.8
	MixSleepNet	1	EEG, EOG, EMG, ECG	83.0	82.1	0.782	89.9	62.5	81.9	89.9	86.0
	SleepFC	10	EEG	79.4	77.8	0.734	88.2	57.3	79.0	86.7	78.0
	ADG-RANet	1	EEG, EOG, EMG, ECG	82.5	81.0	0.775	90.4	61.2	82.6	89.3	81.5
	SleepRWKVNet	1	EEG, EOG, EMG	83.3	81.9	0.790	**90.9**	61.9	82.3	90.5	83.9
	CSPT-SleepNet (Ours)	9	EEG, EOG, EMG	**84.06**	**82.86**	**0.795**	90.5	**62.7**	**83.2**	**91.0**	**87.1**

*Note:* Input Epochs denotes the number of consecutive 30 s epochs used in one forward pass. ‘Multiple’ indicates multi-epoch input without an exact number reported in the original publication. Results for all comparison methods are taken from their corresponding original publications and may involve different channel or modality selections, preprocessing settings, input-context configurations, and evaluation protocols. ‘-’ indicates an unreported or non-comparable result. Bold values indicate the highest reported value within each dataset block.

**Table 3:** Classification results of different sequence supervision strategies on Sleep-EDF-20.

Configuration	Overall Metrics			F1 Score for Each Class (%)				
	ACC (%)	MF1 (%)	κ	W	N1	N2	N3	REM
Equal-weight sequence supervision	86.87 *	82.17 *	0.820 *	92.9	53.5	89.1	88.0	87.3
Center-only sequence supervision	86.80 *	82.31 *	0.819 *	93.1	54.0	88.9	87.8	87.7
Center-driven sequence supervision	**87.47**	**83.08**	**0.829**	**93.3**	**55.9**	**89.4**	**88.1**	**88.8**

*Note:* All three configurations exclude TRC and its associated auxiliary base-logit loss. Bold values indicate the best result among the three supervision configurations. * indicates a statistically significant difference from Center-driven sequence supervision after Holm correction ($p_{\mathrm{adj}}<0.05$).

**Table 4:** Stage transition results of different sequence supervision strategies on Sleep-EDF-20.

Configuration	TOA (%) ↑	Transition-Event Correspondence		
		Pred./True Ratio → 1	Missed Rate (%) ↓	Spurious Rate (%) ↓
Equal-weight sequence supervision	58.33	**1.033**	41.44	**43.29**
Center-only sequence supervision	60.90	1.310	**33.40**	49.16
Center-driven sequence supervision	**61.68**	1.145	35.93	44.05

*Note:* The Pred./True Ratio is dimensionless. A tolerance of ±2 epochs (±60 s) was used for transition matching. ↑ indicates higher is better, ↓ indicates lower is better, and → 1 indicates that values closer to 1 are better. Bold values indicate the best result among the three supervision configurations.

**Table 5:** Ablation results for model components and multimodal fusion strategies on Sleep-EDF-20.

Configuration	Overall Metrics			F1 Score for Each Class (%)				
	ACC (%)	MF1 (%)	κ	W	N1	N2	N3	REM
Full model	**87.93**	**83.53**	**0.836**	**93.6**	**56.7**	**89.8**	**88.3**	**89.3**
w/o Hierarchical Pyramid Pathway	86.96 *	82.39 *	0.822 *	93.0	54.4	89.1	87.6	87.9
w/o Learnable Inter-Level Downsampling	87.45	83.10	0.829	93.4	55.8	89.4	**88.3**	88.7
w/o Fine-Grained Supplementary Branch	87.32	82.86 *	0.827 *	93.2	55.6	89.3	88.1	88.3
Early Fusion	87.09	82.66	0.824 *	93.1	54.7	89.3	87.9	88.4
Late Representation Fusion	87.26	82.86	0.827 *	93.1	55.3	89.5	88.0	88.5
Attention-Based Fusion (w/o JMR)	87.53	83.08	0.830 *	93.4	56.1	89.4	88.1	88.5
w/o TRC	87.47 *	83.08 *	0.829 *	93.3	55.9	89.4	88.1	88.8

*Note:* Bold values indicate the best result among the configurations in the table. * indicates a statistically significant difference from the Full model after Holm correction ($p_{\mathrm{adj}}<0.05$).

**Table 6:** Ablation results for different input modality combinations on Sleep-EDF-20.

Input Modalities	Overall Metrics			F1 Score for Each Class (%)				
	ACC (%)	MF1 (%)	κ	W	N1	N2	N3	REM
EEG	86.01 *	81.22 *	0.808 *	92.1	51.5	88.9	88.1	85.4
EEG+EMG	86.34 *	81.89	0.815 *	91.8	53.2	89.0	87.9	87.7
EEG+EOG	86.87 *	82.15	0.821 *	92.5	53.3	89.3	88.0	87.8
EEG+EOG+EMG	**87.93**	**83.53**	**0.836**	**93.6**	**56.7**	**89.8**	**88.3**	**89.3**

*Note:* Bold values indicate the best result among the configurations in the table. * indicates a statistically significant difference from the full EEG+EOG+EMG configuration after Holm correction ($p_{\mathrm{adj}}<0.05$).

## Data Availability

The datasets analyzed in this study are publicly available from the repositories. The Sleep-EDF-20 and Sleep-EDF-78 datasets were obtained from https://physionet.org/content/sleep-edfx/1.0.0/ (accessed on 23 September 2026). The ISRUC-S3 dataset was obtained from https://sleeptight.isr.uc.pt (accessed on 23 September 2026). The codes used in this study are not readily available because the codes are still being used in an ongoing follow-up research and contain implementation details related to the unpublished work.

## Supplementary Materials

The following supporting information can be downloaded at: https://www.mdpi.com/article/10.3390/brainsci16101108/s1, Figure S1: Training and validation loss and accuracy curves from a randomly selected cross-validation fold of Sleep-EDF-20; Table S1: Sensitivity of classification performance to different boundary epoch inference strategies; Table S2: Statistical analysis of the ablation experiments on Sleep-EDF-20; Table S3: Pooled confusion matrices and class-wise classification metrics for CSPT-SleepNet on Sleep-EDF-20, Sleep-EDF-78, and ISRUC-S3; Figure S2: Fold-wise performance of CSPT-SleepNet on (a) Sleep-EDF-20, (b) Sleep-EDF-78, and (c) ISRUC-S3; Table S4: Classification performance of sequence supervision configurations in the overall loss-scale control experiment on Sleep-EDF-20; Table S5: Sensitivity analysis of transition matching tolerance; Table S6: Computational complexity of the component ablation, multimodal fusion, modality, and epoch-level configurations; Figure S3: Normalized relative modality weights for different sleep stages on Sleep-EDF-20; Figure S4: Inter-epoch attention and modality weights across sleep stages; Table S7: Occlusion analysis of hierarchical relevance for the representative sample shown in Figure 10.
