# Supplementary material for: CSPT-SleepNet: An Automatic Sleep Staging Framework Integrating Center-Driven Sequence Supervision and Multimodal Pyramid Token Encoding
Source: Brain Sci. 2026 Oct 14;16(10):1108. doi: 10.3390/brainsci16101108 (PMC13702679; doi:10.3390/brainsci16101108)
Supplement: Supplementary file 1 [file brainsci-16-01108-s001.zip › brainsci-4617830-supplementary.pdf]

Supplementary Materials

S1. Training and Validation Convergence

To provide a direct view of the optimization behavior of CSPT-SleepNet, Supplementary Figure S1 presents the training and validation loss and accuracy curves from a randomly selected cross-validation fold of Sleep-EDF-20. This fold is shown as a representative example of the training process under the same optimization settings used throughout the cross-validation experiments. As described in the main manuscript, training was limited to 100 epochs, with early stopping applied when the validation accuracy did not improve for 20 consecutive epochs.

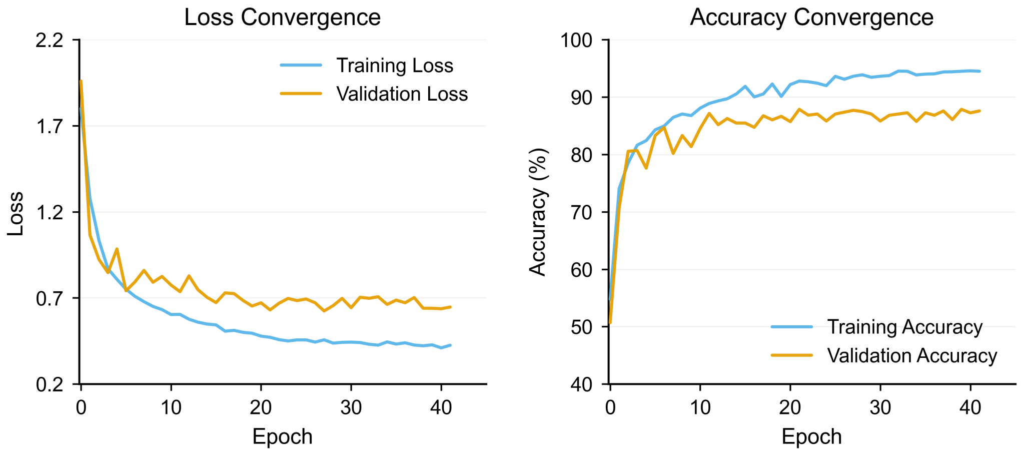

Supplementary Figure S1: Training and validation loss and accuracy curves from a randomly selected cross-validation fold of Sleep-EDF-20.

As shown in Supplementary Figure S1, both the training and validation metrics improve rapidly during the early stage of optimization. The validation loss and accuracy subsequently enter a relatively stable range, indicating that the model reaches a stable convergence state during training. Together with the adopted early-stopping strategy, these curves provide a representative illustration of the training and convergence behavior of CSPT-SleepNet.

S2. Sensitivity to Boundary Epoch Handling

To examine whether the treatment of epochs at the beginning and end of each PSG recording affects the reported whole-night performance, we compared three inference strategies. For the nine-epoch input sequence ($S = 9$), boundary epochs were defined as the first four and last four epochs of each independent recording. The default strategy uses the center-position prediction for epochs that can serve as the center of a complete nine-epoch window and uses probability averaging over valid overlapping windows for the boundary epochs. In the boundary-excluded setting, these eight epochs were omitted from metric calculation. In the reflection-padding setting, the missing context at each recording boundary was filled by reflecting neighboring epochs at the sequence level, allowing each boundary epoch to be placed at the center position of a complete nine-epoch input window. All three strategies used the same trained checkpoints, and no model retraining was performed.

Supplementary Table S1: Sensitivity of classification performance to different boundary epoch inference strategies.

Dataset	Boundary Handling	ACC (%)	MF1 (%)	Cohen's κ	Boundary ACC (%)
Sleep-EDF-20	Overlap averaging (default)	87.93	83.53	0.8356	99.39
	Boundary excluded	87.84	83.49	0.8342	–
	Reflection padding	87.92	83.53	0.8355	98.72
Sleep-EDF-78	Overlap averaging (default)	85.81	80.87	0.8037	99.51
	Boundary excluded	85.72	80.85	0.8027	–
	Reflection padding	85.81	80.87	0.8037	99.59
ISRUC-S3	Overlap averaging (default)	84.06	82.86	0.7951	86.25
	Boundary excluded	84.04	82.85	0.7947	–
	Reflection padding	84.05	82.84	0.7949	85.00

Note: Boundary ACC is calculated only over boundary epochs. '–' indicates that Boundary ACC is not applicable because these epochs are omitted in the boundary-excluded setting.

As shown in Supplementary Table S1, the overall performance was consistent across the three boundary handling strategies. Excluding the boundary epochs changed ACC by only 0.09, 0.09, and 0.02 percentage points on Sleep-EDF-20, Sleep-EDF-78, and ISRUC-S3, respectively, relative to the default strategy. Reflection padding produced even smaller changes in the overall metrics, with ACC differing from the default strategy by no more than 0.01 percentage points across the three datasets. MF1 and Cohen's κ showed similarly limited variation.

The boundary-specific evaluation included 312, 1224, and 80 epochs from Sleep-EDF-20, Sleep-EDF-78, and ISRUC-S3, respectively, representing only a small proportion of the complete overnight recordings. In addition, their class distribution was strongly dominated by stage W in the two Sleep-EDF settings, with all 312 boundary epochs in Sleep-EDF-20 and 1220 of 1224 boundary epochs in Sleep-EDF-78 corresponding to stage W. By contrast, the boundary epochs in ISRUC-S3 showed a more heterogeneous stage distribution. This combination of a small sample proportion and the predominance of W at the recording edges may partly explain the limited influence of boundary handling on the overall performance. Overall, these results indicate that the reported whole-night performance is not materially affected by the selected boundary epoch handling strategy.

S3. Statistical Analysis of Ablation Experiments

Statistical comparisons were based on fold-wise results from Sleep-EDF-20 obtained using identical subject splits, with raw p-values calculated using two-sided paired t-tests. For analyses involving multiple related comparisons, Holm correction was applied separately to ACC, MF1, and Cohen's κ. Statistical significance was defined as a Holm-adjusted p-value below 0.05. Supplementary Table S2 reports both the raw and Holm-adjusted p-values.

For the sequence supervision analysis, Equal-weight and Center-only supervision were compared with Center-driven weak auxiliary supervision. For the component and feature-fusion analyses, the corresponding Full Model was used as the reference. The MPTE component variants, feature-fusion variants, and modality configurations were treated as separate comparison families. Since the TRC analysis involved only one comparison, no multiplicity adjustment was required.

Overall, the statistical results were consistent with the performance trends reported in the main manuscript. For the sequence supervision comparison, the differences between Center-driven supervision and both alternative supervision strategies remained significant for ACC, MF1, and κ after Holm correction. In the component ablations, the statistical evidence varied across architectural modifications and evaluation metrics. Replacing the hierarchical pyramid pathway with parallel multi-scale CNNs and removing TRC produced significant differences in all three overall metrics, whereas the effects of

Supplementary Table S2: Statistical analysis of the ablation experiments on Sleep-EDF-20.

Analysis Group	Configuration	ACC		MF1		κ	
		**Raw p**	**Holm p**	**Raw p**	**Holm p**	**Raw p**	**Holm p**
Sequence supervision strategies	Equal-weight sequence supervision	0.0284	**0.0476**	0.0188	**0.0331**	0.0150	**0.0300**
	Center-only sequence supervision	0.0238	**0.0476**	0.0165	**0.0331**	0.0156	**0.0300**
MPTE components	Parallel multi-scale CNN	0.0162	**0.0485**	0.0212	**0.0424**	0.0116	**0.0347**
	w/ MaxPool inter-level downsampling	0.0725	0.0725	0.0672	0.0672	0.0775	0.0775
	w/o Fine Branch	0.0323	0.0647	0.0123	**0.0369**	0.0176	**0.0351**
Feature fusion strategies	Early fusion	0.0387	0.1061	0.0393	0.0987	0.0225	**0.0478**
	Late fusion	0.0354	0.1061	0.0329	0.0987	0.0201	**0.0478**
	w/o JMR	0.0495	0.1061	0.0412	0.0987	0.0159	**0.0478**
Temporal Residual Correction	w/o TRC	0.0462	**0.0462**	0.0325	**0.0325**	0.0477	**0.0477**
Modality configurations	EEG	0.0055	**0.0165**	0.0156	**0.0469**	0.0035	**0.0106**
	EEG+EMG	0.0373	**0.0483**	0.0354	0.0548	0.0301	**0.0462**
	EEG+EOG	0.0242	**0.0483**	0.0274	0.0548	0.0231	**0.0462**

Note: Bold Holm-adjusted p-values indicate statistical significance at $p < 0.05$.

several local modifications were more metric dependent. In the modality analysis, all reduced-modality configurations showed significant differences from the full multimodal configuration in ACC and κ, while the MF1 difference remained significant only for the EEG-only setting.

S4. Class-Wise Precision, Recall, Specificity, and F1 Scores

Specificity (SP) measures the ability of the model to correctly identify samples that do not belong to a given sleep stage. For each class c, the class was treated as the positive class and the other four stages as the negative class, and specificity was calculated as

$$\mathrm{SP}_c = \frac{\mathrm{TN}_c}{\mathrm{TN}_c + \mathrm{FP}_c},$$

where TN_c is the number of samples from the other stages that were correctly classified as not belonging to class c, and FP_c is the number of samples from the other stages that were incorrectly classified as class c. A higher specificity indicates fewer samples from the other sleep stages being incorrectly assigned to class c.

Supplementary Table S3 reports the pooled confusion matrices and class-wise precision, recall, specificity, and F1 scores for the three datasets evaluated in Section 4.1 of the main text. All class-wise metrics are expressed as percentages and were calculated from the pooled test predictions across all cross-validation folds.

Across the three datasets, W, N2, N3, and REM generally showed relatively high precision, recall, and specificity. N1 remained the most challenging stage, with lower precision and recall than the other stages, consistent with the class-wise F1 results reported in the main manuscript. Specificity remained high across all sleep stages, indicating that samples from other stages were generally well distinguished from the target class.

S5. Fold-Wise Performance Across Cross-Validation Folds

To provide a more detailed view of performance variability across the subject-independent cross-validation partitions, Supplementary Figure S2 presents the fold-wise ACC, MF1, and Cohen's κ for Sleep-EDF-20, Sleep-EDF-78, and ISRUC-S3. The reported values were calculated independently from the test predictions of each fold. For Sleep-EDF-20 and ISRUC-S3, each test fold corresponds to one

Supplementary Table S3: Pooled confusion matrices and class-wise classification metrics for CSPT-SleepNet on Sleep-EDF-20, Sleep-EDF-78, and ISRUC-S3.

**Dataset**	**True Label**	**Predicted Label**					**Class-Wise Metrics (%)**			
		**W**	**N1**	**N2**	**N3**	**REM**	**PR**	**RE**	**SP**	**F1**
	W	7588	529	62	29	77	95.7	91.6	99.0	93.6
	N1	262	1653	527	7	355	54.6	59.0	96.5	56.7
**Sleep-EDF-20**	N2	55	591	15656	965	532	91.7	88.0	94.2	89.8
	N3	4	7	386	5305	1	84.1	93.0	97.3	88.3
	REM	24	246	447	2	6998	87.9	90.7	97.2	89.3
	W	62092	3201	398	44	216	94.1	94.1	97.0	94.1
	N1	3336	11215	5488	83	1400	59.0	52.1	95.5	55.3
**Sleep-EDF-78**	N2	316	3307	61791	2371	1347	85.4	89.4	91.6	87.3
	N3	9	21	2469	10533	7	80.7	80.8	98.6	80.7
	REM	211	1263	2234	26	22101	88.2	85.5	98.2	86.8
	W	1508	128	29	2	7	90.8	90.1	97.8	90.5
	N1	118	741	230	1	127	64.5	60.9	94.5	62.7
**ISRUC-S3**	N2	21	201	2209	155	30	81.9	84.4	91.8	83.2
	N3	3	8	191	1814	0	91.9	90.0	97.6	91.0
	REM	10	70	37	1	948	85.3	88.9	97.8	87.1

Note: Rows denote ground-truth labels and columns denote predicted labels. PR, RE, SP, and F1 denote precision, recall, specificity, and F1 score, respectively.

held-out subject, whereas each test fold of Sleep-EDF-78 contains multiple held-out subjects according to the 10-fold evaluation protocol.

The fold-wise results show the variation in overall classification performance across the subject-independent test partitions. Although some differences are observed among folds, the three overall metrics remain within relatively consistent ranges across the evaluated partitions. These fold-level distributions complement the pooled performance metrics reported in the main manuscript and provide additional information on performance variability across cross-validation folds.

S6. Control Analysis of Overall Loss Scale

To further isolate the effect of asymmetric supervision weighting from that of the overall loss scale, we conducted an additional control experiment on Sleep-EDF-20. In the original Center-driven sequence supervision configuration, the total objective is the sum of the center-epoch loss and the non-center auxiliary loss weighted by $\lambda_{\text{nc}} = 0.3$. Because the non-center loss is averaged across the eight non-center epochs, the sum of the supervision coefficients is 1.3, compared with 1.0 for Equal-weight and Center-only sequence supervision.

To control for this difference, we introduced a normalized Center-driven configuration with the following training objective:

$$\mathcal{L}_{\text{CD,norm}} = \frac{\mathcal{L}_{\text{center}} + \lambda_{\text{nc}}\mathcal{L}_{\text{nc}}}{1 + \lambda_{\text{nc}}}, \qquad \lambda_{\text{nc}} = 0.3. \tag{S1}$$

This normalization reduces the sum of the supervision coefficients to 1.0 without changing the relative weighting between the center and non-center objectives. The three configurations in Supplementary Table S4 used the same model architecture, subject-independent cross-validation splits, and training and evaluation settings, with TRC and its associated auxiliary base-logit loss disabled.

As shown in Supplementary Table S4, the normalized Center-driven configuration maintained higher ACC, MF1, and κ than both Equal-weight and Center-only supervision under the same total supervision coefficient of 1.0. Two-sided paired t-tests were conducted using the same 20 subject-independent folds, with Holm correction applied separately to the two comparisons for each metric. The differences remained statistically significant in both comparisons, with adjusted p-values of 0.0487, 0.0434, and

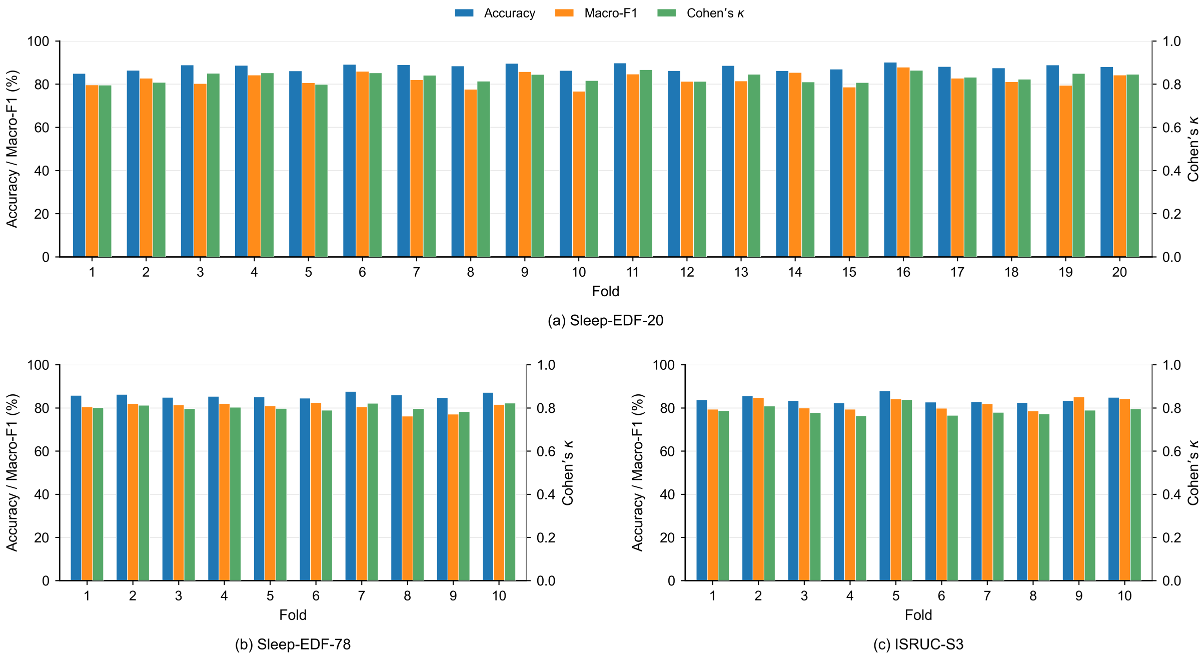

Supplementary Figure S2: Fold-wise performance of CSPT-SleepNet on (a) Sleep-EDF-20, (b) Sleep-EDF-78, and (c) ISRUC-S3. ACC and MF1 are shown on the left vertical axis, and Cohen's κ is shown on the right vertical axis.

Supplementary Table S4: Classification performance of sequence supervision configurations in the overall loss-scale control experiment on Sleep-EDF-20.

**Configuration**	**Weight sum**	**Overall Metrics**			**F1 score for each class (%)**				
		**ACC (%)**	**MF1 (%)**	κ	**W**	**N1**	**N2**	**N3**	**REM**
Equal-weight	1.0	86.87^{*}	82.17^{*}	0.820^{*}	92.9	53.5	89.1	**88.0**	87.3
Center-only	1.0	86.80^{*}	82.31^{*}	0.819^{*}	93.1	54.0	88.9	87.8	87.7
Center-driven (normalized)	1.0	**87.40**	**82.95**	**0.829**	**93.3**	**55.6**	**89.4**	**88.0**	**88.5**

Note: Weight sum denotes the sum of the epoch-wise supervision coefficients. Bold values indicate the highest displayed value for each metric, including ties after rounding. * indicates a statistically significant difference from Center-driven (normalized) sequence supervision after Holm correction ($p_{\mathrm{adj}} < 0.05$).

0.0378 for ACC, MF1, and κ, respectively, in each comparison. These results support the effectiveness of asymmetric supervision weighting under a matched total supervision coefficient, indicating that the observed performance advantage cannot be explained solely by the larger overall loss scale of the original objective.

S7. Sensitivity Analysis of Transition Matching Tolerance

The transition analysis in the main manuscript uses a tolerance of ± 2 epochs for one-to-one matching between predicted and reference transition points when calculating Missed Rate and Spurious Rate. Since each epoch corresponds to 30 s, this setting allows a maximum temporal deviation of 60 s between matched transition events. To examine the robustness of the transition analysis to this predefined tolerance, we further evaluated the supervision strategies using alternative matching ranges of ± 1 and ± 3 epochs.

The matching tolerance only affects whether predicted and reference transition events are considered matched when calculating Missed Rate and Spurious Rate. In contrast, Transition Onset Accuracy (TOA) directly evaluates whether the first epoch after a reference transition is correctly classified, and the Pred./True ratio is determined only by the numbers of detected and reference transitions. Therefore,

both metrics remain identical under different matching tolerances, and only the metrics affected by the tolerance adjustment are presented in this sensitivity analysis.

Supplementary Table S5: Sensitivity analysis of transition matching tolerance.

Tolerance	Method	Missed Rate	Spurious Rate
±1 epoch	Equal-weight	47.21%	48.88%
	Center-only	40.41%	54.51%
	Center-driven	41.70%	49.09%
±2 epochs	Equal-weight	41.44%	43.29%
	Center-only	33.40%	49.16%
	Center-driven	35.93%	44.05%
±3 epochs	Equal-weight	38.96%	40.89%
	Center-only	29.18%	45.93%
	Center-driven	32.55%	41.10%

As shown in Supplementary Table S5, increasing the matching tolerance reduces both Missed Rate and Spurious Rate because a wider temporal range allows more predicted and reference transition events to be matched. However, the relative trends among the evaluated supervision strategies remain consistent across different tolerance settings. These results indicate that the transition behavior observed in the main manuscript is not dependent on the specific choice of the ±2-epoch matching range.

S8. Computational Complexity of Ablation and Reduced Configurations

To complement the component ablation, multimodal fusion, and modality contribution analyses in the main manuscript, we further quantified the computational complexity of the corresponding model variants in terms of trainable parameters and floating-point operations (FLOPs). For the component ablations, the remaining branches were configured to preserve the feature dimensions required by the subsequent processing stages. Therefore, removing or replacing an individual component does not necessarily result in a proportional reduction in parameters or computation. For all sequence-based configurations, FLOPs were measured for one forward pass with a batch size of 1 and a nine-epoch input sequence ($S = 9$). The reported MFLOPs therefore represent the computational cost of processing the complete nine-epoch window. The epoch-level reference processes a single 30 s epoch per forward pass.

As shown in Supplementary Table S6, the computational effects of the component ablations were not uniform because several variants involved structural reconfiguration rather than simple module removal. Replacing the hierarchical pyramid pathway with parallel multi-scale CNNs slightly reduced the parameter count but increased FLOPs. Removing the fine-grained supplementary branch resulted in a small increase in both parameters and FLOPs because the channel capacity originally assigned to this branch was redistributed among the three main-scale branches to preserve the feature dimensions required by subsequent processing. Removing JMR had only a negligible effect on model size and computation, whereas removing TRC produced a more noticeable reduction in the number of trainable parameters. The fusion strategies also showed different complexity profiles. Early Fusion substantially reduced both parameter count and FLOPs, whereas Late Representation Fusion and Attention-Based Fusion had computational costs closer to that of the full model. Thus, the performance differences among the latter configurations mainly reflect differences in the organization of multimodal interaction rather than large differences in model scale.

Reducing the number of input modalities resulted in clearer reductions in computational cost. The EEG-only configuration required 1.760 M parameters and 135.5 MFLOPs, compared with 3.418 M parameters and 397.1 MFLOPs for the full EEG+EOG+EMG configuration. The epoch-level reference was further reduced to 0.542 M parameters and 43.9 MFLOPs for a single-epoch forward pass. Together with

Supplementary Table S6: Computational complexity of the component ablation, multimodal fusion, modality, and epoch-level configurations.

Configuration	Parameters (M)	MFLOPs
Full model	3.418	397.1
w/o Hierarchical Pyramid Pathway	3.362	557.8
w/o Learnable Inter-Level Downsampling	3.411	395.6
w/o Fine-Grained Supplementary Branch	3.423	398.3
Early Fusion	1.170	138.1
Late Representation Fusion	3.266	393.1
Attention-Based Fusion (w/o JMR)	3.418	397.1
w/o TRC	3.293	394.8
EEG	1.760	135.5
EEG+EMG	2.589	267.5
EEG+EOG	2.589	267.5
EEG+EOG+EMG	3.418	397.1
Epoch-level reference	0.542	43.9

the classification results reported in the main manuscript, these measurements provide a complementary view of the trade-off between the additional representation and contextual modeling used by the full framework and the lower computational requirements of reduced configurations.

S9. Stage-Specific Modality Weight Analysis

To further examine the relative allocation of information across modalities in the full model, we computed stage-specific modality weights derived from cross-modal attention. For each test fold, the mean relative weights of EEG, EOG, and EMG at the center position were calculated separately for each ground-truth stage of the center epoch. The fold-level results were then averaged with equal weight, as shown in Supplementary Figure S3. These statistics characterize the average relative allocation of information across modalities at different sleep stages and are analyzed together with the modality ablation results in the main text.

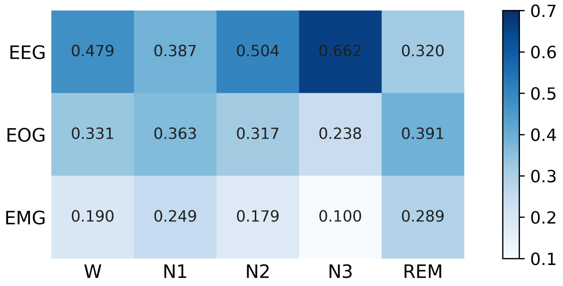

Supplementary Figure S3: Normalized relative modality weights for different sleep stages on Sleep-EDF-20.

As shown in Supplementary Figure S3, modality weights varied across sleep stages. EEG dominated W, N2, and N3, whereas EOG and EMG received greater relative emphasis in N1 and REM than in the other three stages. The EEG weight reached 0.662 in N3, consistent with the importance of high-amplitude slow-wave EEG activity for N3 identification. The modality ablation in the main text shows only limited improvement in N3 after adding EOG and EMG, further supporting the dominant contribution of EEG in the full model for this stage [5,33]. N1 commonly occurs during the transition from wakefulness to

sleep, with relatively gradual EEG changes and possible slow eye movements [5,9]. In REM, the EOG weight reached 0.391, exceeding the EEG weight of 0.320, while EMG also remained relatively high, showing greater relative allocation to EOG and EMG alongside EEG. This distribution is compatible with the physiological characteristics of REM, including rapid eye movements, reduced muscle tone, and low-amplitude mixed-frequency EEG [5,19,33]. It also aligns with the modality ablation results, in which adding EOG improved N1 and REM recognition, while the EEG+EOG+EMG input achieved the best overall performance.

S10. Cross-Stage Inter-Epoch Attention and Modality Weight Analysis

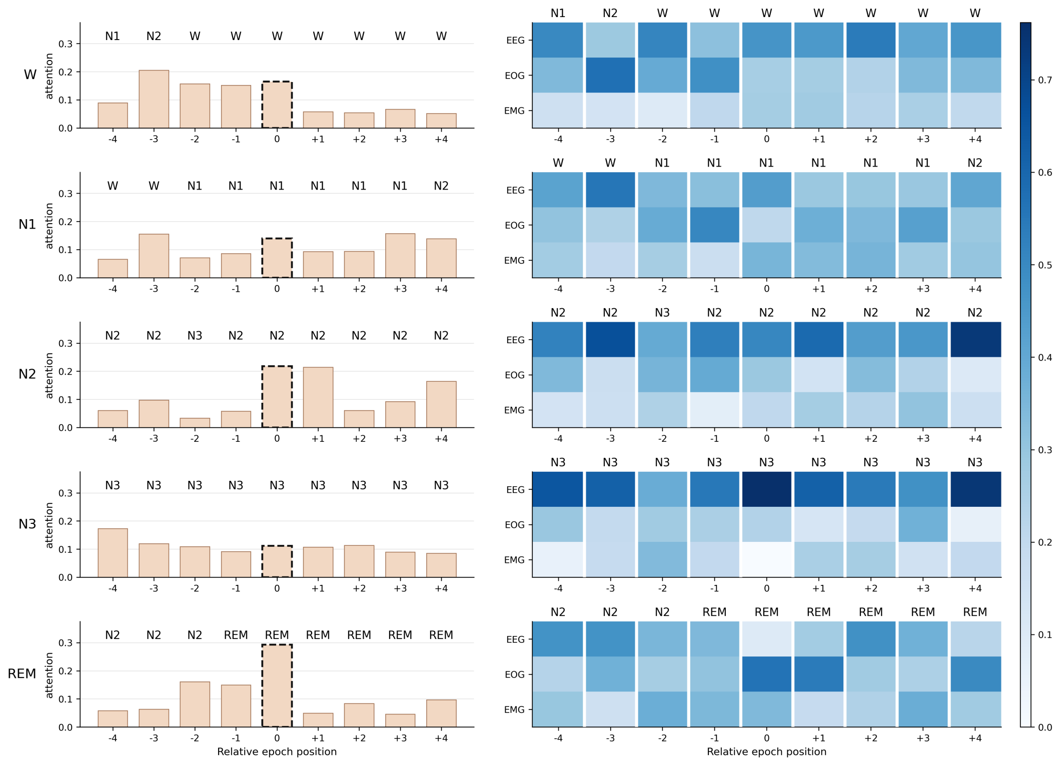

Supplementary Figure S4: Inter-epoch attention and modality weights across sleep stages. The left column shows attention from the center epoch to the nine input positions, and the right column shows the relative weights of EEG, EOG, and EMG at each position. Relative position 0 denotes the center epoch, and the ground-truth sleep stage of the corresponding epoch is marked above each position.

To complement the representative hierarchical analysis in the main text, Supplementary Figure S4 presents correctly classified examples with center epochs from all five sleep stages. The figure shows inter-epoch attention from the center epoch to the nine input positions together with the relative EEG, EOG, and EMG weights at each position.

As shown in Supplementary Figure S4, examples from different sleep stages exhibit distinct inter-epoch attention patterns. The attention distributions vary with the center-epoch stage and its surrounding stage context, involving both same-stage epochs and neighboring stages. In the W example, relatively high attention appears at the preceding N2 epoch and several neighboring W epochs, including the center epoch, with additional attention to the preceding N1 epoch. Here, the attention covers both the transition into W and its subsequent continuation. In the N1 example, attention is distributed across preceding W epochs, consecutive N1 epochs, and the following N2 epoch. These positions include both same-stage N1 context and the neighboring W–N1–N2 progression around the center epoch. In the N2 example, attention is concentrated on the center and neighboring N2 epochs, whereas the intervening N3 epoch

receives less attention. For N3, attention is spread across consecutive N3 epochs, with relatively high weights at several surrounding same-stage positions. The REM example assigns the highest attention to the center REM epoch, while adjacent REM epochs and a preceding N2 epoch also receive relatively high attention.

Relative modality weights within each position also vary with stage and context. Among the examples shown, EEG is consistently dominant in the N2 and N3 examples and receives relatively high weights at most positions in the W example. In the N1 example, the relative weights of EEG and EOG vary across positions. In the REM example, EOG and EMG also receive substantial relative weights at several positions. These patterns are consistent with the modality complementarity reported in the main text.

S11. Occlusion Analysis of Hierarchical Relevance

Supplementary Table S7 provides additional quantitative results of the occlusion experiment described in Section 4.8 for the representative sample shown in Figure 10. Results are summarized for local matched occlusion using 1.0-s, 1.5-s, and 2.0-s windows and for cumulative Top-1, Top-3, and Top-5 occlusion.

Supplementary Table S7: Occlusion analysis of hierarchical relevance for the representative sample shown in Figure 10.

**Analysis**	**Window**	**Mean $\lvert\Delta P\rvert_{\text{high}}$**	**Mean $\lvert\Delta P\rvert_{\text{low}}$**	**Mean High–Low**	**High $>$ Low**
Local	1.0 s	0.001060	0.000242	0.000819	26/27
	1.5 s	0.001050	0.000352	0.000697	25/27
	2.0 s	0.001558	0.000438	0.001120	24/27
Cumulative	1.0 s	0.010431	0.004001	0.006430	3/3
	1.5 s	0.009034	0.004989	0.004044	3/3
	2.0 s	0.015173	0.005570	0.009603	3/3

Note: $|\Delta P|$ denotes the absolute change in the center-stage probability after occlusion. Local results are based on 27 matched comparisons across nine context positions and three modalities, whereas cumulative results are averaged over the Top-1, Top-3, and Top-5 settings. High $>$ Low denotes the number of comparisons in which high-relevance occlusion produced a larger $|\Delta P|$ than the corresponding matched low-relevance occlusion.

As shown in Supplementary Table S7, high-relevance regions generally produced larger changes in the center-stage probability than matched low-relevance regions across the three occlusion lengths. This relationship was observed in 26/27, 25/27, and 24/27 local comparisons for the 1.0-s, 1.5-s, and 2.0-s windows, respectively. The cumulative analysis showed the same pattern across all Top-1, Top-3, and Top-5 settings. The mean perturbation magnitude was also consistently greater for high-relevance occlusion than for matched low-relevance occlusion under all examined settings.
